# Genome assembly of *Polygala tenuifolia* provides insights into its karyotype evolution and triterpenoid saponin biosynthesis

**DOI:** 10.1093/hr/uhad139

**Published:** 2023-07-11

**Authors:** Fanbo Meng, Tianzhe Chu, Pengmian Feng, Nan Li, Chi Song, Chunjin Li, Liang Leng, Xiaoming Song, Wei Chen

**Affiliations:** State Key Laboratory of Southwestern Chinese Medicine Resources, School of Basic Medicine, Chengdu University of Traditional Chinese Medicine, Chengdu 611137, China; >State Key Laboratory of Southwestern Chinese Medicine Resources, Innovative Institute of Chengdu University of Traditional Chinese Medicine, Chengdu University of Traditional Chinese Medicine, Chengdu 611137, China; >State Key Laboratory of Southwestern Chinese Medicine Resources, Innovative Institute of Chengdu University of Traditional Chinese Medicine, Chengdu University of Traditional Chinese Medicine, Chengdu 611137, China; State Key Laboratory of Southwestern Chinese Medicine Resources, School of Basic Medicine, Chengdu University of Traditional Chinese Medicine, Chengdu 611137, China; School of Life Sciences, North China University of Science and Technology, Tangshan 063210, China; >State Key Laboratory of Southwestern Chinese Medicine Resources, Innovative Institute of Chengdu University of Traditional Chinese Medicine, Chengdu University of Traditional Chinese Medicine, Chengdu 611137, China; Institute of Herbgenomics, Chengdu University of Traditional Chinese Medicine, Chengdu 611137, China; School of Life Sciences, North China University of Science and Technology, Tangshan 063210, China; >State Key Laboratory of Southwestern Chinese Medicine Resources, Innovative Institute of Chengdu University of Traditional Chinese Medicine, Chengdu University of Traditional Chinese Medicine, Chengdu 611137, China; Institute of Herbgenomics, Chengdu University of Traditional Chinese Medicine, Chengdu 611137, China; School of Life Sciences, North China University of Science and Technology, Tangshan 063210, China; State Key Laboratory of Southwestern Chinese Medicine Resources, School of Basic Medicine, Chengdu University of Traditional Chinese Medicine, Chengdu 611137, China; >State Key Laboratory of Southwestern Chinese Medicine Resources, Innovative Institute of Chengdu University of Traditional Chinese Medicine, Chengdu University of Traditional Chinese Medicine, Chengdu 611137, China; School of Life Sciences, North China University of Science and Technology, Tangshan 063210, China

## Abstract

*Polygala tenuifolia* is a perennial medicinal plant that has been widely used in traditional Chinese medicine for treating mental diseases. However, the lack of genomic resources limits the insight into its evolutionary and biological characterization. In the present work, we reported the *P. tenuifolia* genome, the first genome assembly of the Polygalaceae family. We sequenced and assembled this genome by a combination of Illumnina, PacBio HiFi, and Hi-C mapping. The assembly includes 19 pseudochromosomes covering ~92.68% of the assembled genome (~769.62 Mb). There are 36 463 protein-coding genes annotated in this genome. Detailed comparative genome analysis revealed that *P. tenuifolia* experienced two rounds of whole genome duplication that occurred ~39–44 and ~18–20 million years ago, respectively. Accordingly, we systematically reconstructed ancestral chromosomes of *P. tenuifolia* and inferred its chromosome evolution trajectories from the common ancestor of core eudicots to the present species. Based on the transcriptomics data, enzyme genes and transcription factors involved in the synthesis of triterpenoid saponin in *P. tenuifolia* were identified. Further analysis demonstrated that whole-genome duplications and tandem duplications play critical roles in the expansion of P450 and UGT gene families, which contributed to the synthesis of triterpenoid saponins. The genome and transcriptome data will not only provide valuable resources for comparative and functional genomic researches on Polygalaceae, but also shed light on the synthesis of triterpenoid saponin.

## Introduction

*Polygala tenuifolia* (2n = 2x = 38), known as ‘Yuan Zhi’ in China, is a perennial herb indexed by the medical pharmacopoeia. As an important medicinal plant, it has been medically used to treat depression, anxiety, insomnia, Parkinson’s disease, and Alzheimer’s disease with a history of more than 2000 years [1, 2]. Saponins, xanthones, and oligosaccharide esters are the main active ingredients of *P. tenuifolia* [3]. The saponins of *P. tenuifolia* had been demonstrated to have protective potential for the central nervous system, such as anti-neuroinflammatory [4], anti-neuronal apoptosis [5], and promote neuronal proliferation [6]. These medical values have led to an increasing commercial demand for *P. tenuifolia*. Hence, *P. tenuifolia* faces the danger of resource depletion [7], and has been regarded as one of the Grade-III Key State-Protected Wild Medicinal Species in China.

*P. tenuifolia* belongs to the Polygalaceae family, which includes about 21 genera and 1000 species. At present, however, no genome of a species in this family has been sequenced. The genome assembly of an increasing number of species allows us to gain a clearer understanding of paleo-polyploidization events and species evolution [8]. Comparative genomics approaches offered the possibility of hierarchical deconstruction of complex genomes affected by recurrent polyploidy [9]. Moreover, using the telomere-centric genome repattern [10], chromosomal evolutionary trajectories can be well reconstructed, which may provide strong evidence for the construction of appropriate phylogenetic relationships [11]. Multi-omics conjoint analysis offers a promising approach for identifying candidate genes responsible for specific biological features [12] and unraveling the biosynthesis of secondary metabolisms [13–15].

The saponins in *P. tenuifolia* are oleanane-type pentacyclic triterpenoids [16]. The synthesis of oleanane-type triterpenoid saponins is initiated from the cyclization of 2,3-oxidosqualene through the catalyzation of *β*-amyrin synthase (*βAS*) and forms *β*-amyrin [17]. Oxidized by cytochrome P450-dependent monooxygenases (P450s) and glycosylated by UDP-glucosyltransferases (UGTs) or cellulose synthase-like (CSLs) enzymes [18], *β*-amyrin finally forms different kinds of saponins [17]. Although the *β*AS [19] and P450 genes [20] have been identified in *P. tenuifolia*, other enzymes in the pathway of saponin synthesis in *P. tenuifolia* were not reported owing to the deficiency of genomic information.

In the present work, a combination of Illumina, PacBio high fidelity (Hifi), and high-throughput chromosome conformation capture (Hi-C) technologies was used to obtain the high-quality *P. tenuifolia* genome, which is the first genome of the Polygalaceae family. By performing comparative genomics analysis, we deciphered the whole genome duplication (WGD) events in *P. tenuifolia* and inferred the evolutionary trajectory of its chromosomes. Combining with transcriptome data, we filtered out a series of genes in the pathways of saponin synthesis in *P. tenuifolia*. These results will not only provide insights into the genomic evolution of *P. tenuifolia*, but also shed light on the synthesis of saponin.

## Results

### Genome sequencing, assembly, and annotation

**Figure 1:**
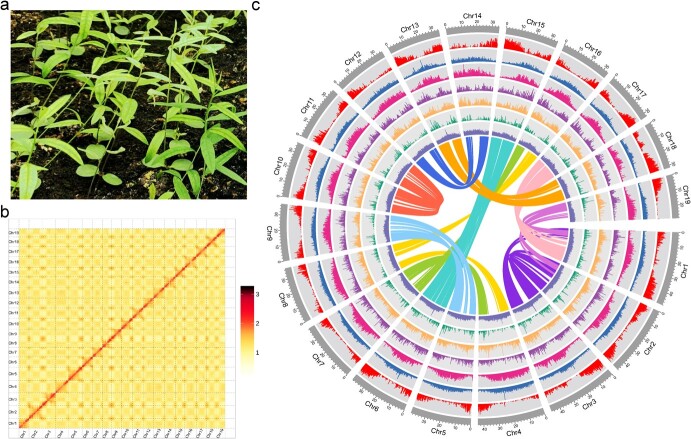
The morphology, Hi-C contact map, and genome features of the *Polygala tenuifolia* genome. **a** The morphology of *P. tenuifolia.***b** Interactions of the 19 pseudochromosomes obtained by Hi-C in the *P. tenuifolia* genome. **c** Genomic features of *P. tenuifolia*. All ratios were calculated using the sliding window size of 200 Kb with no overlapping region. I, chromosome; II to XIII is the distribution of genes, tandem repeat, Gypsy, Copia, DNA repeats, LINE, and, GC ratio, respectively. The curved lines inside the circles linked the colinear genes in the genome.

To estimate the size of *P. tenuifolia* genome (Fig. 1a), we performed preliminary sequencing using Illumina Novoseq 6000 (Illumina, San Diego, CA, USA), and obtained 43.40 Gb data. Results of *K*-mer distribution analysis (*K* = 17, depth = 31) indicated that the size of the *P. tenuifolia* genome was approximately 798 Mb, with the heterozygosity and repeat content of 0.96% and 61.62%, respectively (Fig. S1 and Table S1, see online supplementary material). By combining Illumina, PacBio HiFi and Hi-C technologies, we obtained a reference genome of *P. tenuifolia*. In total, 43.40 Gb of Illumina reads, 24.77 Gb of PacBio HiFi reads, and 43.31 Gb of Hi-C data were generated, resulting in ~31.02× coverage of *P. tenuifolia* genome (Tables S2–S6, see online supplementary material). The assembled sequences were then anchored to 19 pseudochromosomes ranging from 29.22 to 48.17 Mb (Fig. 1b; Table S7, see online supplementary material). The final size of the assembled genome is 769.62 Mb, consisting of 1105 scaffolds (Table 1; Table S8, see online supplementary material). The sequences in the pseudochromosomes account for 92.68% of the genomic sequence (Table S9, see online supplementary material). The GC content of the *P. tenuifolia* genome was 37.32% (Table 1; Table S10, see online supplementary material).

**Table 1:** Statistics of *Polygala tenuifolia* genome assembly and annotation

Genomic feature	Value
Estimated genome size	798.58 Mb
Assembled genome size	769.62 Mb
Sequence assigned to pseudochromosomes	713.30 Mb
Number of contigs	4583
Number of contigs in pseudochromosomes	3497
Longest contig	4 398 726 bp
N50 of scaffolds	330.63 Kb
N50 of contigs	330.63 Kb
GC content	37.32%
BUSCO	93.90%
CEGMA	95.56%
Repeat sequences	60.49%
Number of protein-coding genes	36 643
Genes in pseudochromosomes	35 228
Number of noncoding RNAs	17 221

The accuracy and completeness of the assembled *P. tenuifolia* genome were verified by using short-read sequence alignment, Benchmarking Universal Single-Copy Orthologs (BUSCO), and Core Eukaryotic Genes Mapping Approach (CEGMA). The mapping and coverage rate of all Illumina short reads to the genome were about 96.22% and 98.04%, respectively (Table S11 and Fig. S2, see online supplementary material). Meanwhile, we identified 4 233 765 heterozygous SNPs and 4151 homology SNPs with ratios of 0.679171% and 0.000666%, respectively (Table S12, see online supplementary material). BUSCO analysis showed that 93.90% of the conserved genes belonging to the complete and single-copy, complete and duplicated, fragmented, and missing categories, accounting for 1727 (74.2%), 458 (19.7%), 22 (0.9%), and 119 (5.2%) of the total genes, respectively (Fig. S3 and Table S13, see online supplementary material). In addition, CEGMA analysis confirmed that 237 genes (95.56%) were assembled from 248 core eukaryotic genes (Table S14, see online supplementary material), indicating that the assembly is relatively complete.

It was found that 60.49% of the *P. tenuifolia* genome are repetitive sequences, with long terminal repeats (LTRs) accounting for 58.88% (Fig. S4, Table S15 and S16, see online supplementary material). By integrating the predictions of *ab initio* gene prediction, homologous identification, and transcripts derived from three tissues, we annotated 36 643 protein-coding genes in the *P. tenuifolia* genome (Fig. 5 and Table S17, see online supplementary material). The average length of predicted coding sequences is 1127 bp with 4.85 exons (Table 1). Approximately 93.41% of all annotated genes were homologous to proteins in well-known protein databases. For example, 92.56% to the proteins in the Non-Redundant Protein Sequence (NR) database, 91.02% to the proteins in InterPro database, 74.07% to the proteins in Swiss-Prot database, and 68.23% to the proteins in Kyoto Encyclopedia of Genes and Genomes (KEGG) database (Fig. 1c; Table S18, see online supplementary material). We also annotated the noncoding RNAs in *P. tenuifolia* and identified 607 miRNAs, 14 559 rRNAs, 1385 snRNAs, and 670 tRNAs (Table S19, see online supplementary material).

### Gene family evolution

**Figure 2:**
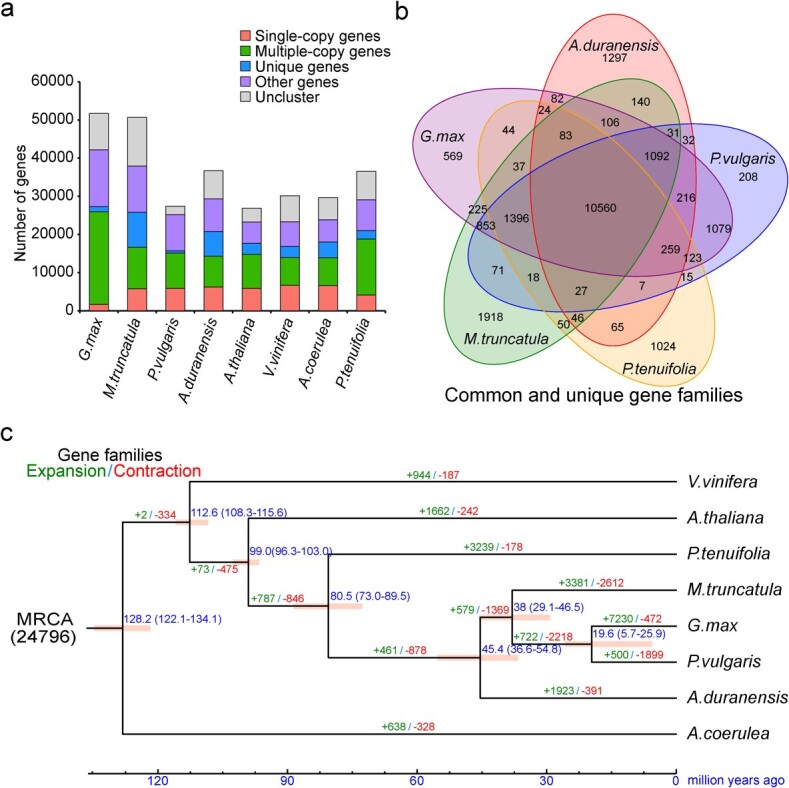
Gene family analyses. **a** Number of single-copy, multi-copy, unique, and other genes in *Polygala tenuifolia* and the other seven species. **b** Common and unique gene families in *P. tenuifolia* and four legume species. **c** Gene family expansion/contraction. Green and red numbers depict the number of expanded and contracted gene families, respectively. The blue numbers around evolutionary nodes represent the divergence time of the species (million years ago, Mya).

We compared the *P. tenuifolia* genome with genomes of the other seven plants, namely four Fabaceae species (*Arachis duranensis*, *Medicago truncatula*, *Phaseolus vulgaris*, and *Glycine max*), *Aquilegia coerulea* from early-diverging eudicotyledons, *Arabidopsis thaliana*, and *Vitis vinifera* (Table S20, see online supplementary material). Based on gene family cluster analysis, a total of 289 738 genes from the eight species were clustered into 24 802 families, of which 822 single-copy gene families were shared by these species (Fig. 2a). Further analysis demonstrated that 10 560 families were shared by the five Fabales species, and 1024 families including 2569 genes were specific to *P. tenuifolia* (Fig. 2b). Gene ontology (GO) enrichment analysis of these 2569 genes revealed that they were enriched in transferase activity, monooxygenase activity, oxidoreductase activity, and cellular ion homeostasis (Table S21, see online supplementary material). Meanwhile, by KEGG enrichment analysis, we found that these genes were mainly enriched in pathways involved in flavonoid biosynthesis, phenylpropanoid biosynthesis, and stilbenoid, diarylheptanoid and gingerol biosynthesis (Table S22, see online supplementary material).

By analysing the expanded and contracted gene families in the above mentioned eight species, we found that their most recent common ancestor (MRCA) had 24 796 gene families. Compared with MRCA, 3239 gene families (containing 10 909 genes) in *P. tenuifolia* genome had expanded and 178 gene families (containing 375 genes) had contracted (Fig. 2c). GO and KEGG enrichment analysis showed that these expanded gene families were mainly involved in the regulation of squalene monooxygenase activity, cellulose synthase (UDP-forming) activity, UDP-glucosyltransferase activity, and flavonoid biosynthesis (Tables S23 and S24, see online supplementary material), whereas the contracted gene families were involved in plant-pathogen interaction, isoflavonoid biosynthesis, and terpene synthase activity (Tables S25 andS26, see online supplementary material).

### Two rounds of whole genome duplication

To decipher the evolutionary history of the *P. tenuifolia* genome, we first identified intra- and inter-genomic gene collinearity of the eight genomes. We identified 23 220 gene pairs from 1174 homologous blocks in the *P. tenuifolia* genome, which is less than the 68 270 gene pairs from the 2118 homologous blocks found in the *G. max* genome, but more than those in other analysed genomes (Table S27, see online supplementary material). The longest collinear region was identified between chromosomes 6 and 15, which contained 243 gene pairs. Similar to the *G. max* genome, there are more colinear gene pairs and more complete colinear regions in the *P. tenuifolia* genome than in other genomes, suggesting that one or more additional WGD events may have occurred in the *P. tenuifolia* genome.

**Figure 3:**
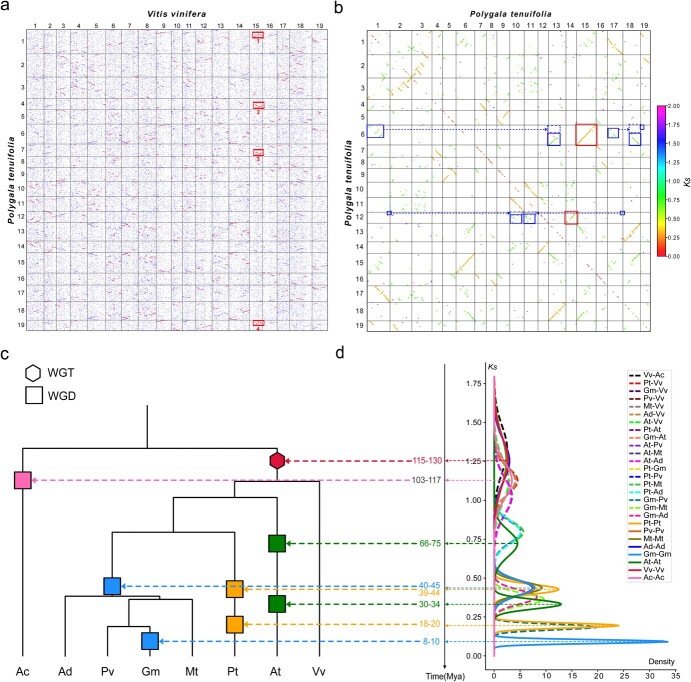
Dot plots of homologous genes and WGD dating. **a** The homologous genes dot plots between *Polygala tenuifolia* and *Vitis vinifera*. The best-hit, second-best-hit, and other hit genes are indicated by red, blue, and gray dots, respectively. The red box demonstrates an example of the orthologous ratio of 1:4 between *V. vinifera* and *P. tenuifolia*. **b** Dot plots of colinear genes and *Ks* within *P. tenuifolia* genome. The color of the dot indicates the *Ks* of the gene pair. Red and blue boxes indicate the colinear gene regions produced by different WGDs, respectively. **c** Species phylogenetic tree for *P. tenuifolia* (Pt), *Aquilegia coerulea* (Ac), *Arachis duranensis* (Ad), *Medicago truncatula* (Mt), *Phaseolus vulgaris* (Pv), *Glycine max* (Gm), *Arabidopsis thaliana* (At), and *V. vinifera* (Vv). **d** Corrected number of *Ks* between colinear genes of intra (solid lines) and inter genomes (dashed lines).

We created a series of homologous gene dot plots, which are helpful in locating paralogs within species and orthologs between different species. In the dot plot between *V. vinifera* and *P. tenuifolia* (Fig. 3a), we found four best-matched chromosome regions in *P. tenuifolia* for almost every grape chromosome (orthologous ratio 1:4), which is similar to the pattern in the dot plot between grape and *G. max* (Fig. S6, see online supplementary material). In the dot plot within the *P. tenuifolia* genome, we found that each *P. tenuifolia* chromosome had one best-matched chromosomal region with two sub-matched regions (Fig. 3b; Fig. S7, see online supplementary material). These results suggested that *P. tenuifolia* experienced two WGD events. In addition, we found that the orthologous ratio was 4:2 between *P. tenuifolia* and *M. truncatula* or *P. vulgaris* (Figs. S8 and S9, see online supplementary material), and 4:4 between *P. tenuifolia* and *G. max* (Fig. S10, see online supplementary material), indicating the independent WGD history between *P. tenuifolia* and legume species.

We further estimated the synonymous substitution rates at synonymous sites (*Ks*) of collinear gene pairs within and between genomes, and calculated the distribution of the median *Ks* in the collinear region. The *Ks* density curve within the *P. tenuifolia* genome showed three peaks at 1.90 (+/−0.206), 0.66 (+/−0.049), and 0.30 (+/−0.024) (Fig. S11 and Table S28, see online supplementary material), suggesting that it experienced a core eudicot-common hexaploidy (ECH) event and two sequential WGD (hereinafter referred to as Pt-*β* and Pt-*α*, respectively) events, respectively. By aligning the *Ks* peaks generated by the same evolutionary event (Fig. S12, see online supplementary material), we corrected the *Ks* generated by all events (Table S29, see online supplementary material). Based on previous reports of ECH that occurred ~115–130 million years ago (Mya), we inferred that the Pt-*β* and Pt-*α* events occurred ~39–44 and ~18–20 Mya, respectively (Fig. 3c and d). Accordingly, the time divergence of *P. tenuifolia* and legumes was estimated to be 73–83 Mya, which is close to the phylogenetically inferred dates based on the MCMCtree (Fig. S13, see online supplementary material).

### Gene loss and genome fractionation

**Figure 4:**
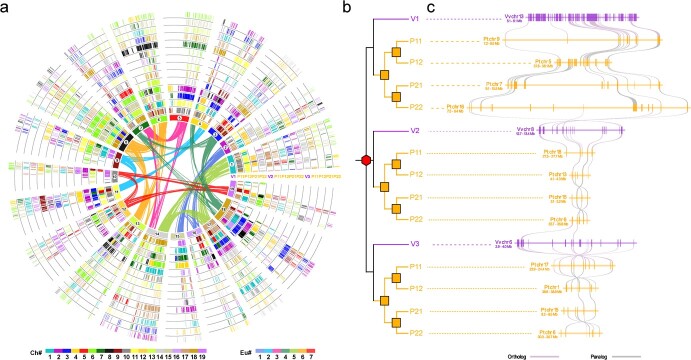
Global and local alignment of *Polygala tenuifolia* and *Vitis vinifera* subgenomes. **a** Global alignment of colinear genes between *P. tenuifolia* subgenomes (P11, P12, P21, P22) and *V. vinifera* subgenomes (V1, V2, V3). The chromosomes of *V. vinifera* are in the innermost circle, and the colinear gene pairs generated by ECH events are connected by curves with the same color as the ancestral chromosomes (Eu). The homologous regions between *V. vinifera* and *P. tenuifolia* genomes form the other circles in which genes are represented by short lines corresponding to the color of the chromosome (Ch). **b** A putative phylogenetic gene tree for *V. vinifera* and *P. tenuifolia*. Red hexagons indicate gamma events occurred in core eudicots evolution; yellow squares indicate tetraploid events occurred in *P. tenuifolia* evolution. **c** Local alignment of genes among *V. vinifera* and *P. tenuifolia* (Pt).

A list of collinear genes between the grape and *P. tenuifolia* genomes was constructed (Fig. 4a; Table S30, see online supplementary material). In the absence of gene loss or translocation, there would be four *P. tenuifolia* orthologs (P11, P12, P21, and P22) for one grape gene (V1, V2, or V3) (Fig. 4b). However, it was found that only 265 (0.94%) grape genes had four *P. tenuifolia* orthologs, indicating the large-scale genome fractionation of the *P. tenuifolia* genome. Chromosomal regions generated by the Pt-*β* and Pt-*α* events have divergent gene retention levels (Fig. S14, see online supplementary material). We selected a local fragment of the alignment to show the changes in numbers of genes (Fig. 4c). The 1 Mb region of grape chromosome 13 (from 5.1 to 6.1 Mb) had 72 genes, while only 13, 26, 19, and 14 genes were retained in the homologous region of *P. tenuifolia* chromosomes 9, 5, 7, and 16, respectively. Similar results were also observed for the regions from chromosome 8 and chromosome 6 (Fig. 4c), respectively. In summary, the average loss rate of collinear genes in *P. tenuifolia* was 64.24% with reference to grape. For example, compared with the orthologous chromosome 16 in grape, 74.78% of the collinear genes in the *P. tenuifolia* genome were lost (Table S31, see online supplementary material).

We further explored the magnitude and potential mechanisms of gene loss after polyploidization of the *P. tenuifolia* genome. Using the grape genome as a reference, we investigated the successive deletion of genes in the *P. tenuifolia* genome. After excluding the loss of chromosomal segments, we identified 2804 single gene loss cases, while the number of consecutive gene loss cases diminished rapidly (Fig. S15 and Table S32, see online supplementary material). Statistical fitness regression showed that the pattern of consecutive gene loss obeying a near geometric distribution, with an extension parameter of 0.28 with a goodness of fit F-test *P*-value of 0.906 (Table S33, see online supplementary material).

### Ancestral karyotypes and chromosome evolution trajectories

**Figure 5:**
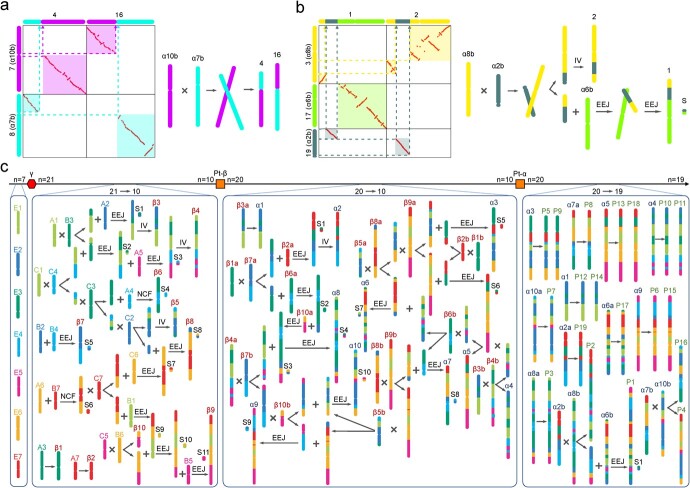
Evolutionary trajectories of the extant *Polygala tenuifolia* chromosomes. X indicates a crossing-over between neighboring chromosomes. + indicates a fusion of two adjacent chromosomes. IV indicates segmental inversion. EEJ indicates end-to-end joining of two chromosomes. NCF indicates nested chromosome fusion of two chromosomes. a and b after a chromosome number indicate that they are duplicated copies. For example, *β*1a and *β*1b are duplicates of *β*1. Satellite chromosomes are indicated by S. The schematic diagram of the evolutionary trajectory of (**a**) chromosomes 4 and 16, and (**b**) chromosomes 1 and 2. The numbers of the horizontal and vertical coordinates represent extant *P. tenuifolia* chromosomes, and the numbers with letters in brackets represent ancestral chromosomes. Chromosomes are represented by rods with different colors and projected to the dot plots. **c** Trajectories of chromosomal karyotype evolution from core eudicot ancestors to extant *P. tenuifolia*. The number of chromosomes is indicated on the top. The red hexagon represents the ECH event. The orange square represents the WGD event. E1–E7 are the ancestral chromosomes before the ECH event. A1–A7, B1–B7, and C1–C7 are the ancestral chromosomes after the ECH event. *β*1-*β*10 are the ancestral chromosomes before the Pt-*β* event. *α*1-*α*10 are the ancestral chromosomes before the Pt-*α* event.

We reconstructed the ancestral chromosomes of the Polygalaceae and inferred their evolutionary trajectory of becoming extant chromosomes (Fig. 5). By analysing the dot plot within the *P. tenuifolia* genome (Fig. 3b), we deduced that it contained 10 chromosomes prior to the Pt-*α* event (pre-Pt-*α* chromosomes, denoted as *α*1-*α*10). As shown in Figure 3b, near-complete collinearity existed between *P. tenuifolia* chromosomes 5 and 9, 6 and 15, 10 and 11, 12 and 14, and 13 and 18, indicating that they were formed by the duplication of five pre-Pt-*α* chromosomes, respectively. The remaining five pre-Pt-*α* chromosomes formed 10 chromosomes after the Pt-*α* event. Subsequently, five of the 10 chromosomes underwent two crossover events and one end–end joining (EEJ) event, and finally formed the four extant chromosomes (Figure 5a and b). Specifically, a crossover between *α*7b and *α*10b caused a reciprocal translocation of chromosome arms (RTA) to form the present chromosome 4 and 16 (Fig. 5a). In addition, a crossover between *α*2b and *α*8b formed the present chromosome 2 and the precursor of chromosome 1. Then, the precursor and *α*6b underwent an EEJ, which formed the present chromosome 1 (Fig. 5b).

By analysing the collinear gene dot plot of the grape and *Buxus sinica* genomes, we identified the 21 chromosomes of the post-γ ancestral genome (Fig. S16, see online supplementary material). The 19 chromosomes of grape were used to reconstruct the 21 ancestral chromosomes (A1-A7; B1-B7; C1-C7) of core eudicot plants, tripled from seven pre-γ ancestral chromosomes: E1-E7 (Fig. 5c;Fig. S17, see online supplementary material). We compared the 10 chromosomes of pre-Pt-*α* with grape chromosomes to infer the karyotype before and after the Pt-*β* event (Fig. S17, see online supplementary material). If two or more post-γ chromosomes show homologous to pre-Pt-*α* ancestral chromosomes twice, these chromosomes should have merged before the Pt-*β* event. If two or more post-γ chromosomes show homologous to pre-Pt-*α* ancestral chromosomes one time, they most likely merged after the Pt-*β* event. However, in fact, the telomere positions of post-γ chromosomes B2 and B4 were joined together in both *α*2 and *α*10 (Fig. 5c;Fig. S17, see online supplementary material), which suggested their fusion into the post-*β*chromosome *β*7 via an EEJ event prior to the Pt-*β* event. One copy of B4 appears as an integrated part in *α*2, while the other was included in both chromosomes *α*9 and *α*10, which could be explained by accidental crossover between the *β*4a and *β*7b followed by a crossover with *β*10b (Fig. 5c).

Taken together, we reconstructed the evolutionary trajectory of *P. tenuifolia* chromosomes, which experienced the Pt-*β* and Pt-*α* events (Fig. 5c). Prior to the Pt-*β* event, 21 post-γ chromosomes experienced nine EEJs, two nested chromosome fusions (NCFs) and six crossovers, forming 10 pre-Pt-*β* chromosomes (*β*1-*β*10). Before the Pt-*α* event, 20 post-Pt-*β* chromosomes experienced 10 EEJs and 11 crossovers, forming 10 pre-Pt-*α* chromosomes (*α*1-*α*10). After the Pt-*α* event, 20 post-Pt-*α* chromosomes experienced two crossovers and one EEJ, and finally formed the 19 present chromosomes (P1-P19). During the whole process, two telomeres formed the satellites or B chromosomes (S1-S11) via EEJ or NCF, however, which might be lost during evolution and resulted in a reduced number of chromosomes.

### Genes involved in regulating the triterpene saponins biosynthesis

**Figure 6:**
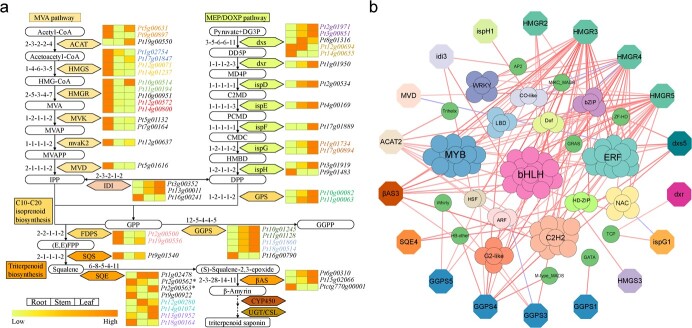
Genes involved in the triterpene saponins biosynthesis. **a** Genes related to triterpene saponins biosynthesis in *Polygala tenuifolia*. The genes were identified from *Arabidopsis thaliana*, *P. tenuifolia*, *Vitis vinifera, Phaseolus vulgaris*, and *Glycine max*. The numbers on the left side indicate the number of genes identified in *A. thaliana*, *P. tenuifolia*, *V. vinifera, P. vulgaris*, and *G. max*, respectively. The heatmap shows the gene expression (TPM) in root, stem, and leaf of *P. tenuifolia*. The neighboring homochromatic genes are colinear genes generated by Pt-*α* events. **b** Correlation analysis of between transcription factors (indicated by circles) and genes involved in triterpene saponins biosynthesis (indicated by octagons). Only the transcription factors and genes with the absolute value of correlation coefficients greater than 0.99 were shown and indicated by lines with different colors. Red and blue represents positive and negative correlation, respectively. The darker color indicates the higher correlation.

The biosynthesis of various triterpene saponins, which are the important medicinal components of *P. tenuifolia*, involves a series of catalytic enzymes. Here, we sought to identify genes participating in the regulation of triterpene saponin synthesis by using genomic and transcriptomic data. Using the 45 sequences of 20 enzymes related to triterpene biosynthesis in *Arabidopsis* as seeds, we performed Blastp and identified their homologs in *P. tenuifolia*, *V. vinifera*, *P. vulgaris*, and *G. max* (Fig. 6a; Table S34, see online supplementary material). These five species had one or more genes in all nodes of the regulatory pathway. The *P. tenuifolia* genome contained 41 genes for terpenoid backbone biosynthesis and 12 genes for triterpenoid biosynthesis, which included three genes encoding *β*AS. Combined with the list of collinear genes (Table S30, see online supplementary material), we found that 13 pairs of genes (26/53, 49.06%) involved in triterpene synthesis were derived from the Pt-*α* event, implying the WGD event plays an important role in triterpene biosynthesis in *P. tenuifolia*.

To explore the expression patterns of triterpene-related genes in *P. tenuifolia*, we performed transcriptome analysis of its three different tissues (root, stem, and leaf). In the MVA pathway, most genes expressed in the roots at higher levels than those in the other two tissues. However, the expression of most genes in the MEP pathway was lower in the roots (Fig. 6a;Table S35, see online supplementary material). Interestingly, some WGD-generated genes had different expression patterns in some tissues, suggesting that their function may be altered after the WGD event. For example, *Pt11g00194* and *Pt10g00514*, encoding Hydroxymethylglutaryl-CoA reductase (HMGR), were a pair of genes produced by Pt-*α*. *Pt11g00194* was highly expressed only in root, whereas *Pt10g00514* was highly expressed in both root and leaf.

The biosynthesis of saponins in *P. tenuifolia* originates from the oxidation and glycosylation of the triterpenoid backbone, which are catalyzed by P450s and UGTs. By using homology search, we identified 248 P450 genes and 152 UGT genes in the *P. tenuifolia* genome. To identify the crucial P450s and UGTs participating in the biosynthesis of oleanane-type saponin backbones, we performed co-expression analysis of the P450 and UGT genes with the *βAS* gene *PtβAS3* (*Ptctg770g00001*), respectively (Figs S18 andS19). It was found that the expression patterns of 14 P450 genes and 15 UGT genes were similar to that of *PtβAS3* (Table S36, see online supplementary material). Phylogenetic analysis showed that two P450 genes were from CYP88A subfamily, two P450 genes were from subfamily CYP93D, four UGT genes belonged to UGT709A1 subfamily, and three UGT genes belonged to UGT91C8 subfamily (Figs S20 andS21, see online supplementary material).

Furthermore, we identified the transcription factors in *P. tenuifolia* and obtained 2544 transcription factors belonging to 57 different families (Table S37, see online supplementary material). Based on their expression correlations, we constructed regulatory networks between genes involved in *P. tenuifolia* saponins biosynthesis pathway and the transcription factors (Fig. 6b). It was found that 18 triterpene backbone genes were closely correlated with 98 transcription factors. These transcription factors belong to 24 families, with the largest number of members being the bHLH family (15 genes), followed by the MYB family (14 genes) (Table S38, see online supplementary material). In addition, we also identified 245 and 188 transcription factors that were closely correlated with the expression patterns of 72 P450 and 48 UGT genes (Fig. 6b; Figs S22 andS23, Tables S39 and S40, see online supplementary material), respectively.

## Discussion

Within the Fabales order, the genomes of more than 50 species in the Fabaceae family have been sequenced, which facilitated the studies of functional genomics of economically important traits [21–23]. However, previous studies on Polygalaceae were only focused on its chloroplast genome [24–26]. In the present work, we successfully assembled the first reference genome of *P. tenuifolia* in the Polygalaceae and sequenced the transcriptomes of different tissues (root, stem, and leaf). Although we only obtained a collapsed assembly, this version of the assembly could be used as a reference for obtaining a high-resolution genome of *P. tenuifolia*. In addition, the reported data will be also valuable resources for functional genetic and evolutionary studies of *P. tenuifolia* and other Polygalaceae.

In the evolutionary history of angiosperms, WGD (or paleopolyploidy) events have made great contributions to plant adaptation during global environmental change [27, 28]. Our comparative genomics analysis showed that *P. tenuifolia* experienced two additional WGD events after its divergence with the Fabaceae family. By analysing the distribution of *Ks* between homologous genes, we estimated that the two WGDs in *P. tenuifolia* occurred at ~39–44 and ~18–20 Mya, respectively. The genomes of newly formed polyploids may undergo rapid loss of homologs and genomic restructuring following polyploidization [29]. Thus, we analysed the rate of colinear gene loss in the *P. tenuifolia* genome and found that it was consistent with a segment-by-segment removal model [30, 31], with gradual accumulation leading to genomic fractionation after polyploidization. The two WGD events and the subsequent genomic instability may provide great opportunities for species divergence, natural selection and new species evolution in the Polygalaceae.

Plants experienced chromosomal rearrangement and reduction after WGD events, and eventually reverted to disomic inheritance [32]. By using the telomere-centered chromosome rearrangement model [10], we inferred the number of chromosomes and chromosome evolutionary trajectories at different evolutionary stages of *P. tenuifolia*. However, this trajectory was rather crude due to the lack of more closely related species for comparison. With the availability of more genomes of Polygalaceae species and closely related species, a more accurate evolutionary scenario of ancestral genomes and Polygalaceae family genomes will be comprehensively inspected. The systematical investigation of karyotype evolution in the *P. tenuifolia* broadens our knowledge about Polygalaceae evolution, provides a valuable example of karyotype analysis in plants that experienced several rounds of whole genome duplication, and could successively facilitate following functional genomics studies, such as the dissection of key enzymes in secondary metabolite biosynthesis.

Combining genomic and transcriptomic analyses, we investigated the genetic profile of the biosynthesis and regulation of saponins, and identified the key genes involved in the processes. Interestingly, we found one *βAS* gene (*Ptctg770g00001*) with high expression in the root tissues, indicating it has a key role in the synthesis of *β*-amyrin in *P. tenuifolia* root, which is a key intermediate in the synthesis of oleanane-type saponins [17, 33]. As biosynthetic pathways were often clustered on chromosomes [34, 35], we identified specialized metabolic pathway genes clustered in the *P. tenuifolia* genome using the PlantiSMASH analysis pipeline [36]. In addition, we also identified a total of 28 potential gene clusters related to secondary metabolism in the *P. tenuifolia* genome (Table S41, see online supplementary material), of which only one gene cluster (Cluster 25) belongs to the terpene cluster and is likely to be involved in *P. tenuifolia* saponins biosynthesis, as it contained a *β*AS gene (*Pt6g00310*). Except for this gene, none of the relevant enzymes in the triterpene saponin synthesis pathway were included in any gene cluster. However, four gene clusters (Cluster 13, 16, 20, and 24) contained UGT and P450 genes, suggesting a tendency for UGT and P450 genes to be existed in clusters, which may be related to the synthesis of *P. tenuifolia* saponins. In addition, we investigated the duplication patterns of the P450 and UGT genes (Tables S42 andS43, see online supplementary material). The P450 family had 87.5% (217/248) and 5.24% (13/248) genes associated with WGD and tandem replication, respectively. For UGT genes, 67.76% (103/152) genes were found in WGD and 22.37% (34/152) genes in tandem type. As a result, it was hypothesized that tandem duplications and WGD were crucial to the expansion and evolution of the P450 and UGT genes in *P. tenuifolia*.

## Materials and methods

### Genome sequencing, assembly, and quality assessment

We collected leaf samples from *P. tenuifolia* Willd and performed genomic DNA isolation and library construction. To obtain high-quality reference genomes, we combined three different sequencing strategies (Illumina's short reads, PacBio's subreads, and Hi-C's interaction reads). According to standard procedures, we extracted genomic DNA from leaves using the QIAGEN kit. For genome investigation and evaluation, we constructed Illumina genomic libraries in accordance with Illumina's recommended protocol and performed paired-end read (2 × 150 bp) sequencing by using the Illumina NovaSeq 6000 platform. Following the manufacturer's protocol, we used the SMRTbell Express Template Prep Kit 2.0 to create SMRTbell libraries (15-20 K) and then used Pacbio Sequel IIe to obtain high-fidelity (HiFi) reads. Finally, according to the Hi-C protocol [37], the Hi-C libraries were constructed by using the original sample as input. The Hi-C sequencing libraries were amplified by PCR (12–14 cycles) and then sequenced on Illumina PE150.We estimated the genome size of the *P. tenuifolia* based on 17-mer of Illumina sequencing data.

The Hifiasm program (v0.14-r312) [38] with the default parameter was used to perform the assembly of the *P. tenuifolia* genome. The ALLHiC (v0.9.8, with the parameters, -K 19, --minREs 50, --maxlinkdensity 3, and --NonInformativeRabio 0.) program [39] was used to process Hi-C data after alignment control and HiCUP [40] quality control for assisting the *P. tenuifolia* genome assembly. Then, the Juicebox (1.11.08) program [41] was used to visualize the Hi-C data. Finally, we evaluated the assembled genomes using BUSCO (v4.1.2, lineage dataset eudicots_odb10) [42] and CEGMA (V2.5) [43] software with the default parameters. We used the BWA (0.7.8) software [44] to map short reads to the assembled genome and counted the reads' comparison rate, coverage of the genome, and distribution of depth.

### Genome annotation

The homology alignment together with *de novo* search were used to identify the whole genome repeats. For *ab initio* prediction, Tandem Repeats Finder (v4.07b, http://tandem.bu.edu/trf/trf.html) was used to extract tandem repeat sequences from the *P. tenuifolia* genome. The repeat regions in the *P. tenuifolia* genome were extracted using Repbase [45] and RepeatMasker (v4.0.5, with the parameters, −a -nolow -no_is -norna -parallel 4). A new database of repetitive elements was generated by using LTR_FINDER [46], RepeatScout (v1.0.5) [47], and RepeatModeler (v1.0.8, http://www.repeatmasker.org/RepeatModeler.html) with their default parameters. We removed the repetitive sequences with length less than 100 bp and gap ‘N’ greater than 5% to obtain the original transposable element (TE) library, and generated a non-redundant library using uclust processing of the combination of Repbase and our TE library. Finally, based on the above results, repeats at the DNA-level were identified.

We adopted a combined strategy of *ab initio* prediction, homology-based prediction, and RNA-Seq assisted prediction to predict genes. The *ab initio* prediction was performed using Augustus (v3.2.3) [48], Geneid (v1.4), Genescan (v1.0) [49], GlimmerHMM (v3.04) [50], and SNAP (2013-11-29) [51]. For homology-based prediction, protein sequences of the other seven species (*A. duranensis*, *M. truncatula*, *P. vulgaris*, *G. max*, *A. coerulea*, *A. thaliana*, and *V. vinifera*) were collected from phytozome and peanutbase (Table S20, see online supplementary material). Subsequently, Tblastn (E-value ≤1e-5) was employed to align proteins to the *P. tenuifolia* genome. Based on the matched sequences, GeneWise (v2.4.1) software [52] was used to predict the gene structures in *P. tenuifolia*. The RNA-Seq assisted genome annotation was performed using transcriptome reads assemblies generated by Trinity (v2.1.1) [53] with the parameters, --normalize_reads --full_cleanup --min_glue 2 --min_kmer_cov 2 --KMER_SIZE 25. TopHat (v2.0.11) [54] with default parameters was used to align the RNA-Seq reads to *P. tenuifolia* genome to optimize the genome annotation and identify exon regions. Transcript assembly of the genome was obtained by using the default parameters of Cufflinks [55]. Finally, we obtained the non-redundant reference gene set by using EvidenceModeler (EVM, v1.1.1, with the parameters, --segmentSize 200 000 –overlapSize 20 000 --min_intron_length 20) and Program to Assemble Spliced Alignment (PASA) [56].

We used Blastp (E-value ≤1e-5) to align proteins with the Swiss-Prot [57] database and annotated gene function based on the best match. We used InterProScan (v4.8) [58] to search public databases (Pfam, ProDom, PANTHER, SMRT, PRINTS, and PROSITE) to identify the motifs and domains. Then, we assigned the GO ID for each gene according to the InterPro entry. In addition, we mapped gene sets to the KEGG [59] pathway based on the best match for each gene.

The tRNAscan-SE (v1.3.1) [60] program was used to detect tRNAs in the *P. tenuifolia* genome. We chose rRNAs from the above-mentioned seven species as references and predicted rRNAs in *P. tenuifolia* using BLAST (v2.2.26). The Infernal (http://infernal.janelia.org/) was used to search the Rfam database [61] with default parameters to identify microRNAs and snRNAs. Finally, we constructed a Circos plot of the genome Circos using TBtools [62, 63].

### Comparative genomics analysis

The genome of *P. tenuifolia* was compared with those of seven other plants, including *A. coerulea*, *V. vinifera*, *A. thaliana*, *A. duranensis*, *M. truncatula*, *P. vulgaris*, and *G. max*. OthoMCL (v1.4) [64] was used to perform a full protein sequence similarity search (main inflation value of 1.5, other parameters using default settings) to infer orthologous genes in the eight species. Then, we performed multiple sequence alignment of all single-copy gene families separately using MUSCLE (v3.8.31, with default parameters) [65] and combined all the alignments to form a super-alignment matrix. Finally, a phylogenetic tree was constructed based on the maximum likelihood method using RAxML (8.2.12) [66]. The MCMCTree in the PAML v4.9 package [67] was employed to evaluate the divergence time between *P. tenuifolia* and the other seven species. We used the following time scales for calibration: *A. coerulea*–*A. thaliana* (122–134 Mya), *A. thaliana*–*V. vinifera* (105–115 Mya), and *A. thaliana*–*P. vulgaris* (97–109 Mya). The CAFE (v4.2) software [68] (−p 0.05 -t 4 -r 100) was used to perform gene family expansion and contraction analysis.

We identified homologous genes inter- and intra-genome using Blastp (E-value ≤1e-5) [69]. The WGDI (v0.6.1) program [70] integrated an improved version of ColinearScan [71] was used to infer the collinearity of the genome. We calculated *Ks* between colinear genes using the Nei-Gojobori method in the yn00 program of PAML [67]. The dot plots of homologous genes between and within genomes were generated using WGDI (−d and -kd options). We classified colinear genes generated by different events based on the complementarity of colinear regions in the dot plots and the range of colinear gene *Ks*, and then fitted the probability density curves of the median value of *Ks* in colinear blocks using a Gaussian function. Correction of the evolutionary rate was performed using the method reported previously [72, 73].

### Reconstructing ancestral karyotypes of the Polygalaceae

We inferred the number of Polygalaceae ancestral chromosomes and the chromosomal evolutionary trajectories based on the telomere-centric chromosome recombination model [10]. First, we inferred that the ancestor had 10 chromosomes before the Pt-*α* event using the colinear gene dot plots within the *P. tenuifolia* genome. For the two integrally homologous chromosomes, we used the chromosome containing more colinear genes as the ancestral chromosome. Next, we used the colinear gene dot plots between the grape and *B. sinica* genome [74] to infer the 21 ancestral chromosomes of the core eudicot after the ECH event and to locate the approximate positions of their telomeres in the extant genome. Finally, using the homologous gene dot plots of the grape and the pre-Pt-*α* genome, we investigated the positional relationships of the telomeres of the 21 ancestral chromosomes to infer the EEJ and NCF. By combing the occurrence of EEJ and NCF, we inferred that the pre-Pt-*β* ancestor had 10 chromosomes.

### Transcriptome analysis

We collected roots, stems and leaves of *P. tenuifolia* Willd for RNA-Seq analysis (three biological replicates for each tissue), and isolated RNA from tissues using the QIAGEN kit. Sequencing of quality-controlled RNA was performed with Illumina Novaseq 6000. The yielded high quality and clean reads were used for downstream analysis (Table S44, see online supplementary material). We aligned the clean reads to the *P. tenuifolia* genome through the HISAT2 software (v2.0.5) [75] with the default parameters. The novel transcripts were predicted by StringTie software (v1.3.3b) [76] with default parameters. We used the featureCounts tool (v1.5.0-p3) [77] in the subread software to filter out unqualified reads. Finally, the expression value of each gene was represented using TPM. Differentially expressed genes were detected using DESeq2 [78] with |log_2_(FoldChange)| ≥ 1 and adjusted *P*-value ≤0.05. Enrichment analysis of GO and KEGG was performed using the clusterProfiler package [79].

### Identification of genes related to triterpene saponins biosynthesis

We downloaded protein sequences related to triterpene synthesis in *A. thaliana* from the KEGG database. We used Blastp (E-value <1e-5, identify >50%, score > 200) to identify the homologous genes related to triterpene synthesis in *P. tenuifolia* and the four legume plants (*A. duranensis*, *M. truncatula*, *P. vulgaris*, and *G. max*). We identified the P450 and UGT gene families in *P. tenuifolia* based on their HMM profiles (PF00067 and PF00201) using the HmmSearch in the TCMPG [80, 81]. Heatmaps of gene expression were drawn using the heatmap function in the seaborn library of python. The maximum likelihood-based phylogenetic trees were constructed by using MEGA X [82] and visualized by iTOL v5 [83]. The transcription factors in the *P. tenuifolia* genome were identified based on the PlantTFDB database [84]. Based on the expression values, we calculated the Pearson correlation coefficients between transcription factors and genes involved in triterpene synthesis, and visualized the correlation networks using Cytoscape software [85]. The gene duplication types were identified by the gene_classifier_program integrated in the MCScanX package [86].

## Supplementary Material

Web_Material_uhad139

## Data Availability

The genome sequence and RNA-seq data of *P. tenuifolia* were deposited in the Genome Sequence Archive in BIG Data Center at Beijing Institute of Genomics (BIG, Chinese Academy of Sciences), under the accession numbers CRA009096 and CRA009246, which are publicly accessible at http://bigd.big.ac.cn/gsa. The annotations of the *P. tenuifolia* genome can be downloaded from the TCM Plant Genome Database (TCMPG: http://cbcb.cdutcm.edu.cn/TCMPG/resource/genomes/details/?id=TCMPG20196).
