# Supplementary material for: Genome assembly of Polygala tenuifolia provides insights into its karyotype evolution and triterpenoid saponin biosynthesis
Source: Hortic Res. 2023 Jul 11;10(9):uhad139. doi: 10.1093/hr/uhad139 (PMC10476160; doi:10.1093/hr/uhad139)
Supplement: Web_Material_uhad139 [file web_material_uhad139.zip › Supplementary Figures 1-23.pdf]

Supplementary Figures

Chromosome-level Genome Assembly of *Polygala tenuifolia* Provides Insights into Karyotype Evolution and Triterpenoid Saponin Biosynthesis

Fanbo Meng[1,2], Tianzhe Chu[2], Pengmian Feng[1], Nan Li[3], Chi Song[2,4], Chunjin Li[3], Liang Leng[2,4*], Xiaoming Song[3*], Wei Chen[1,2,3*]

[1]State Key Laboratory of Southwestern Chinese Medicine Resources, School of Basic Medicine, Chengdu University of Traditional Chinese Medicine, Chengdu 611137, China;
[2]State Key Laboratory of Southwestern Chinese Medicine Resleources, Innovative Institute of Chengdu University of Traditional Chinese Medicine, Chengdu University of Traditional Chinese Medicine, Chengdu 611137, China;
[3]School of Life Sciences, North China University of Science and Technology, Tangshan 063210, China;
[4]Institute of Herbgenomics, Chengdu University of Traditional Chinese Medicine, Chengdu 611137, China.

*To whom correspondence should be addressed: Liang Leng, lling@cdutcm.edu.cn; Xiaoming Song, songxm@ncst.edu.cn; Wei Chen, greatchen@ncst.edu.cn

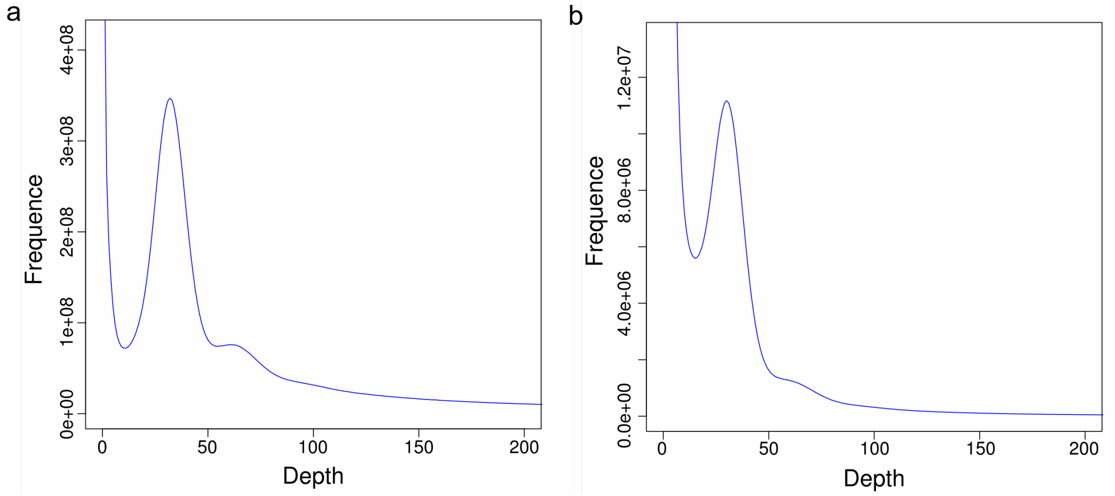

Supplementary Fig. 1 *K*-mer (k =17) analysis for estimating the genome size of *P. tenuifolia*. The horizontal axis is the depth, and the vertical axis is the *K*-mer number (a) and types (b) at the corresponding depth.

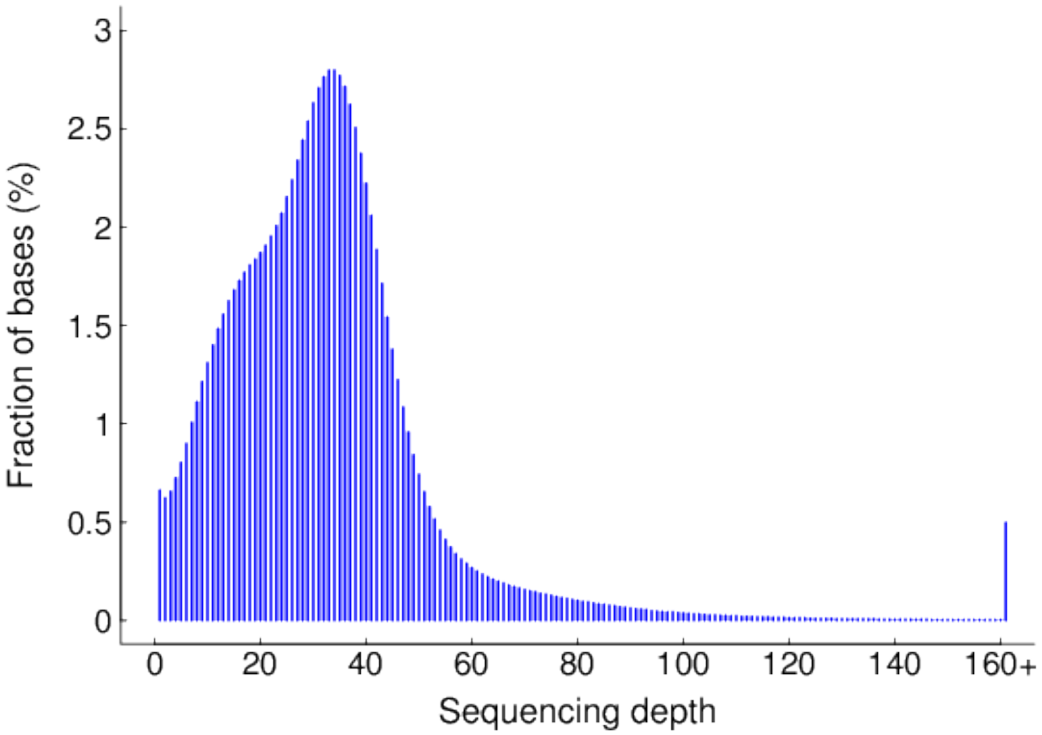

Supplementary Fig. 2 The distribution of sequencing depth of *P. tenuifolia* genome.

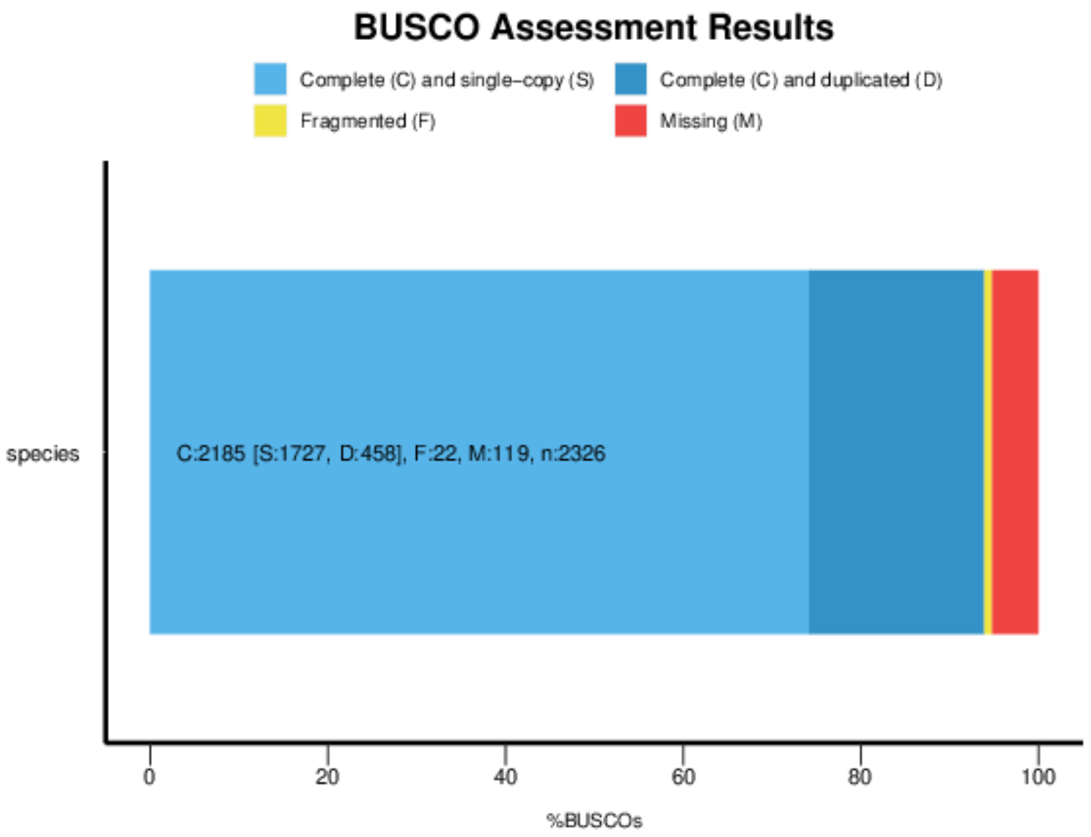

Supplementary Fig. 3 Evaluation of the *P. tenuifolia* genome using the Benchmarking Universal Single-Copy Orthologs (BUSCO) notation.

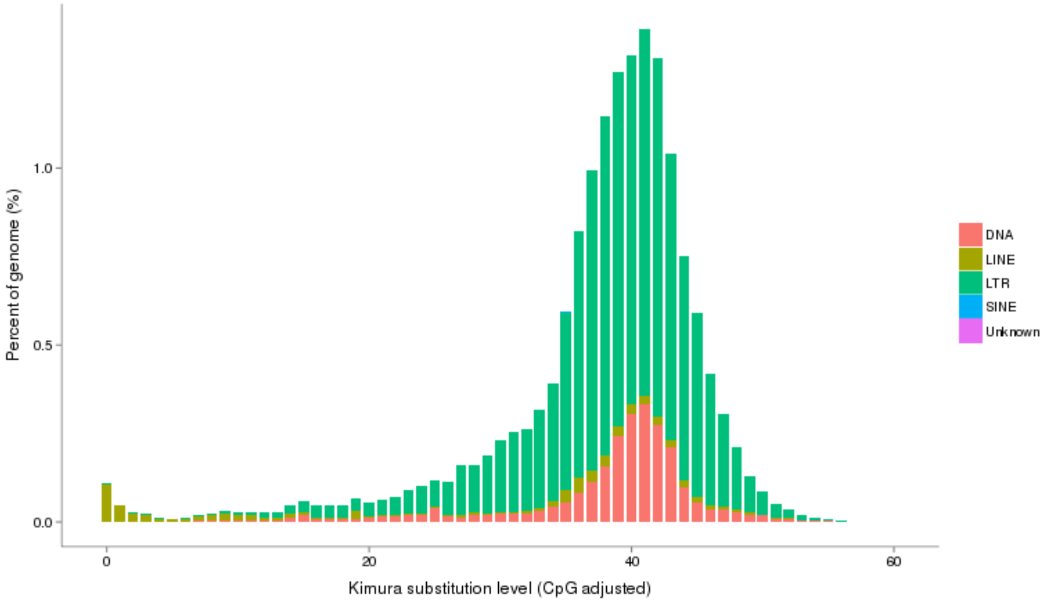

Supplementary Fig. 4 The frequency of the main different types of repetitive sequences in *P. tenuifolia* genome, including DNA, LINE, LTR, and SINE repeats.

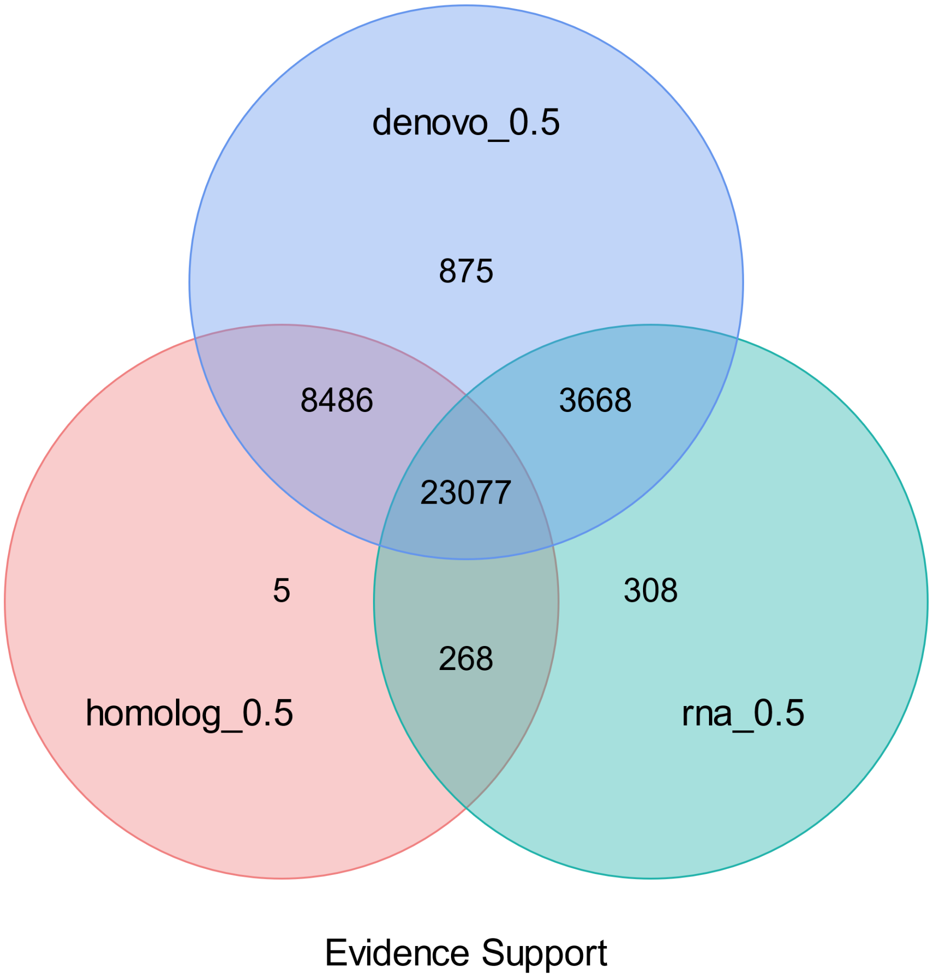

Supplementary Fig. 5 Number of gene structures supported by the three strategies. denovo_0.5, EVM integrates genes supported by Denovo prediction; homolog_0.5, genes supported by homologous prediction when EVM integration; rna_0.5, genes supported by RNA-seq during EVM integration. Supported genes are the ones with overlap >50% against known genes.

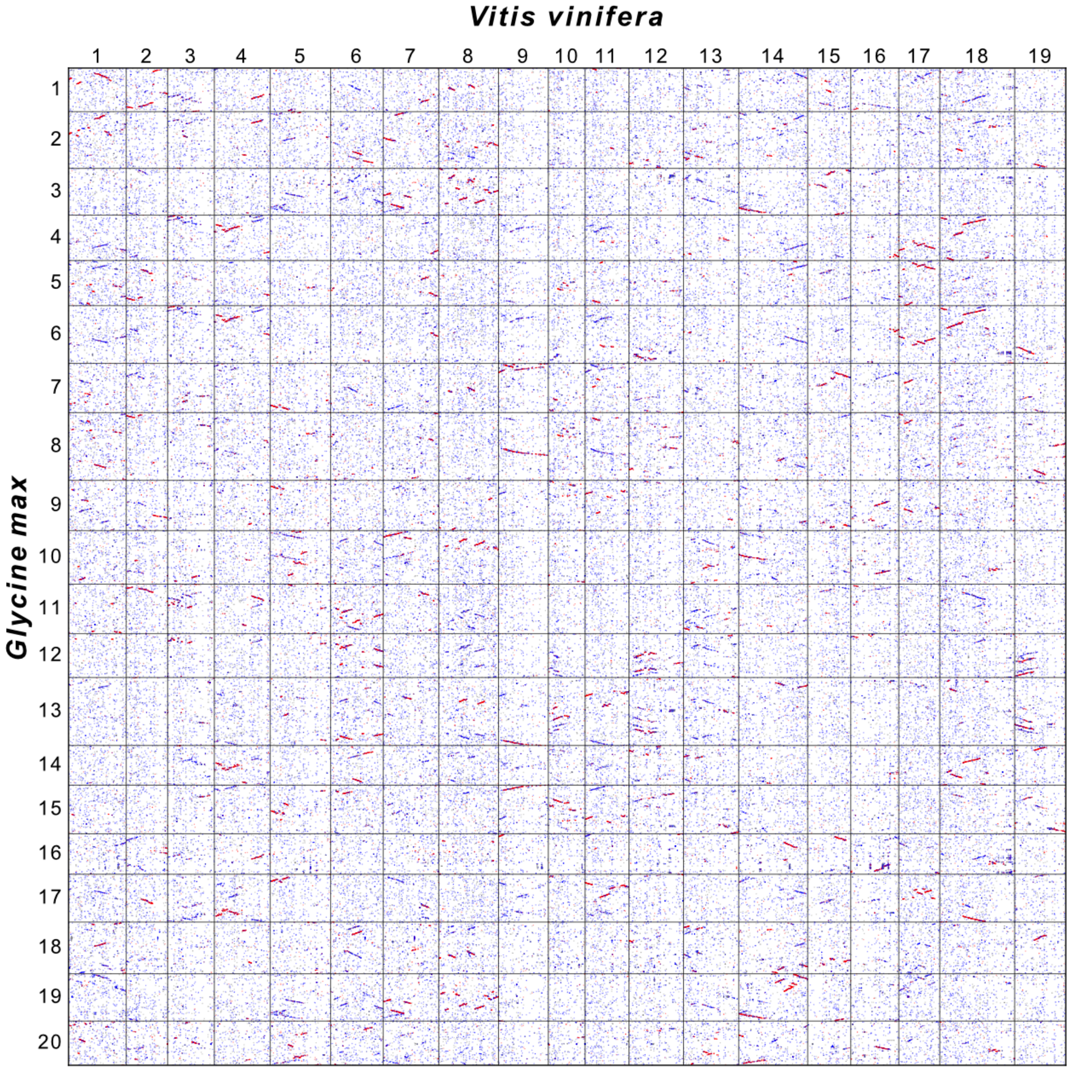

Supplementary Fig. 6 Homologous gene dot-plots between grape and soybean shows an orthologous gene ratio 1:4.

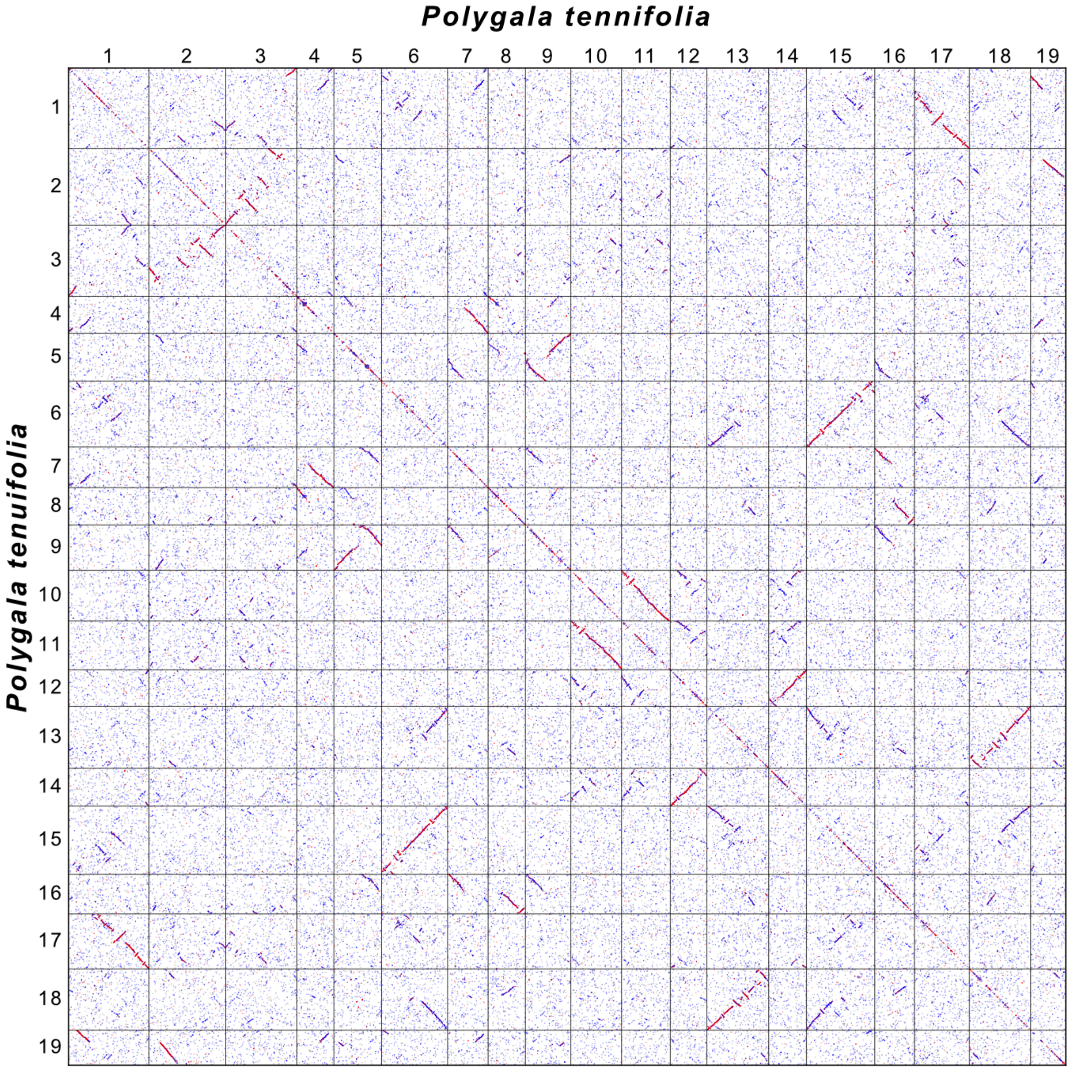

Supplementary Fig. 7 Homologous gene dot-plots within *P. tenuifolia* genome shows an orthologous gene ratio 1:1.

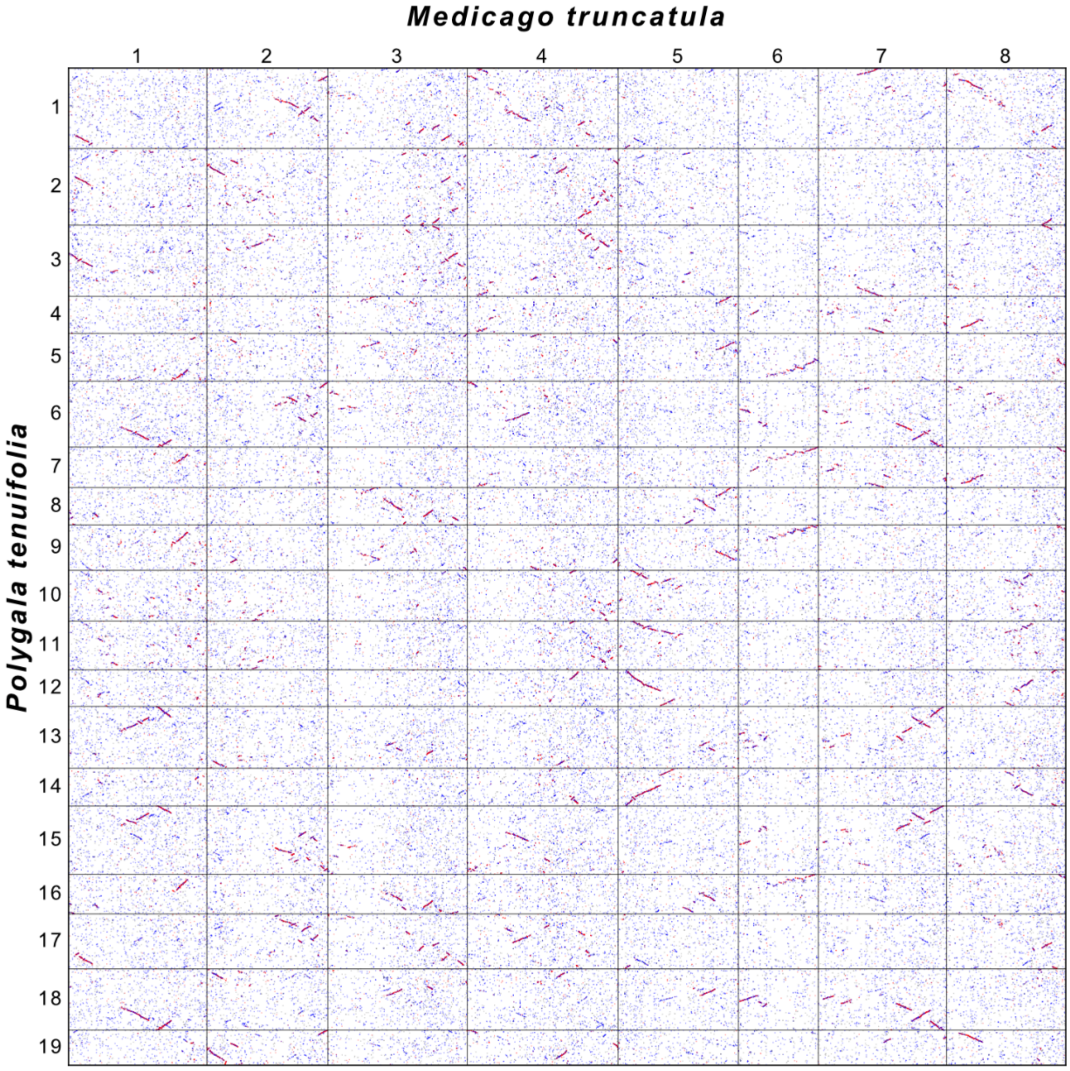

Supplementary Fig. 8 Homologous gene dot-plots between *P. tenuifolia* and *M. truncatula* shows an orthologous gene ratio 4:2.

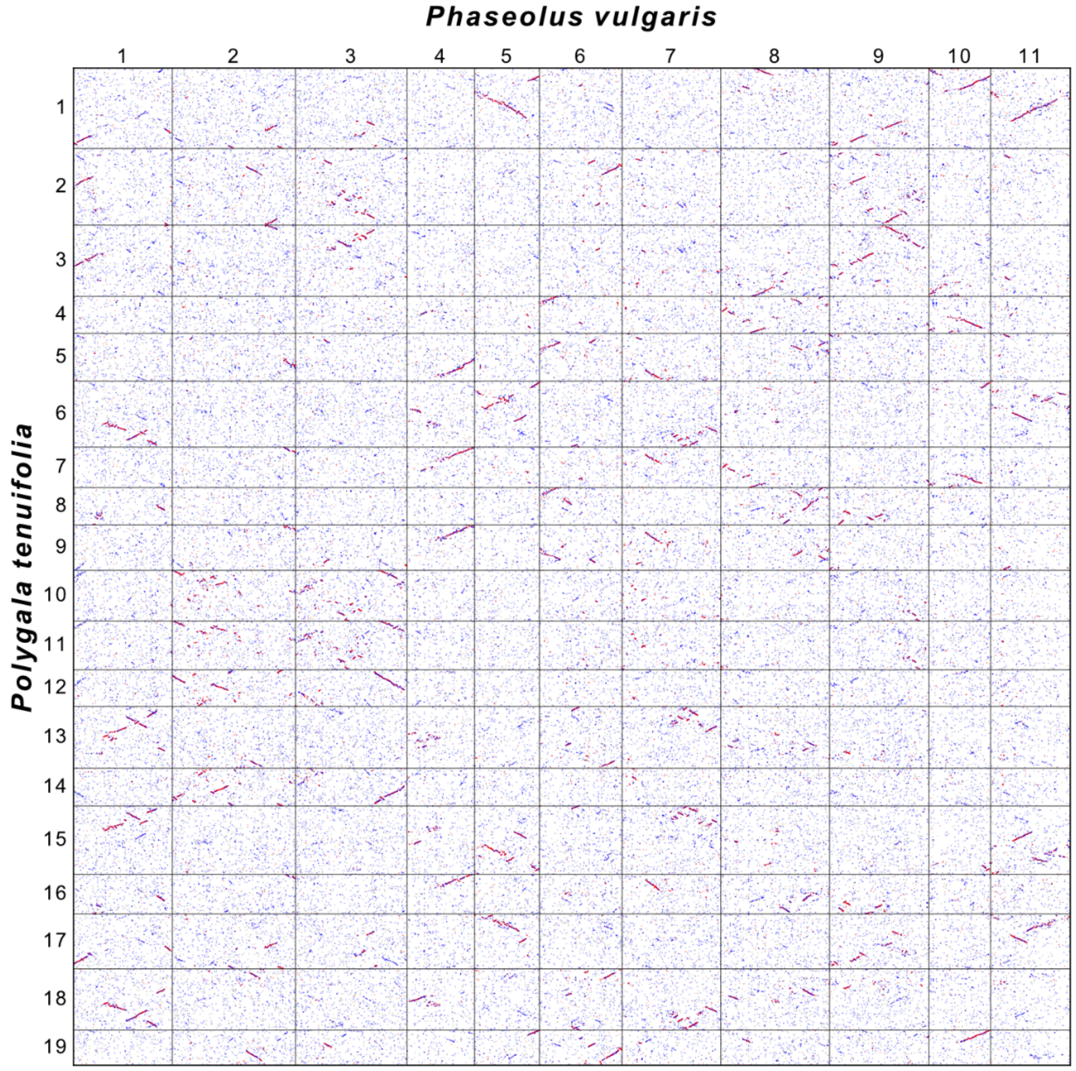

Supplementary Fig. 9 Homologous gene dot-plots between *P. tenuifolia* and *P. vulgaris* shows an orthologous gene ratio 4:2.

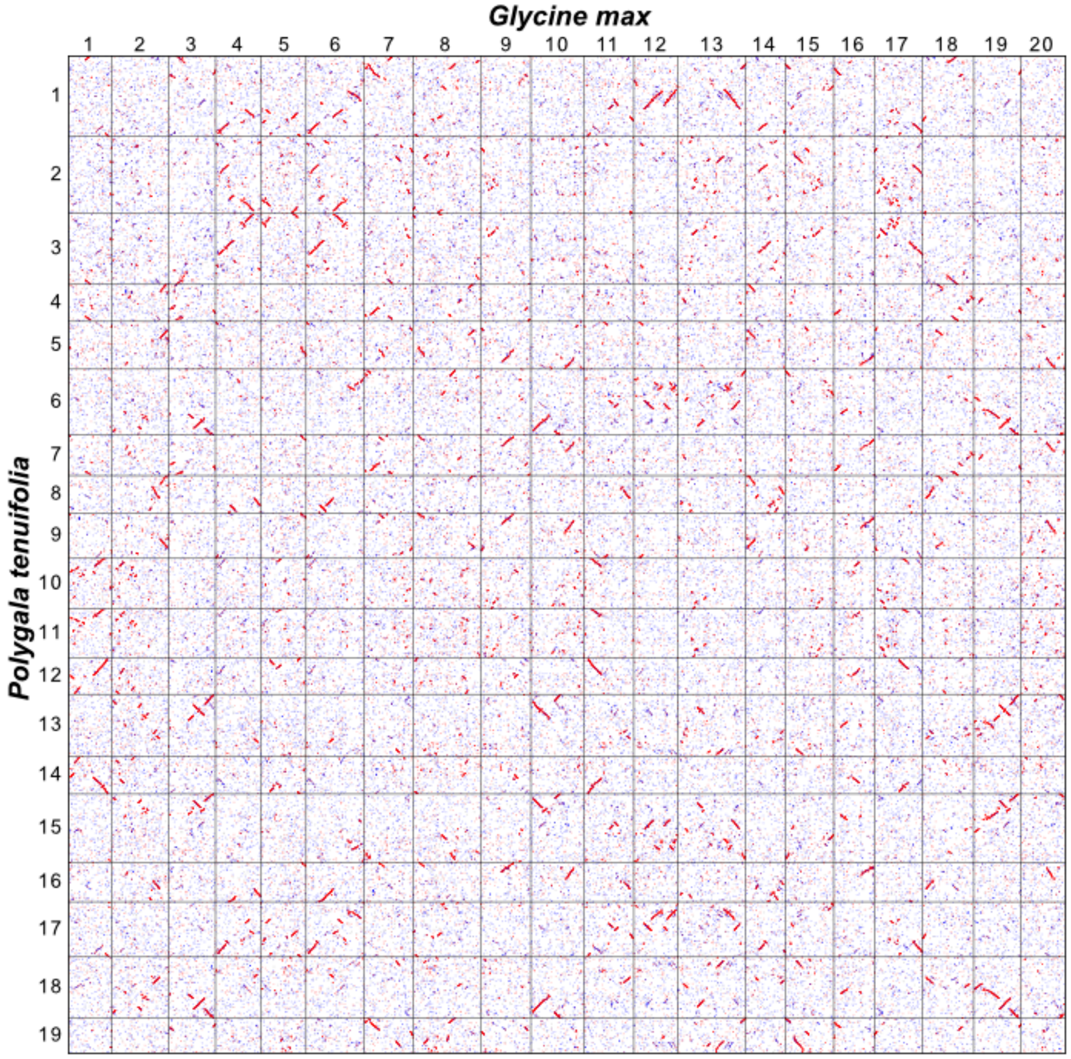

Supplementary Fig. 10 Homologous gene dot-plots between *P. tenuifolia* and *G. max* shows an orthologous gene ratio 4:4.

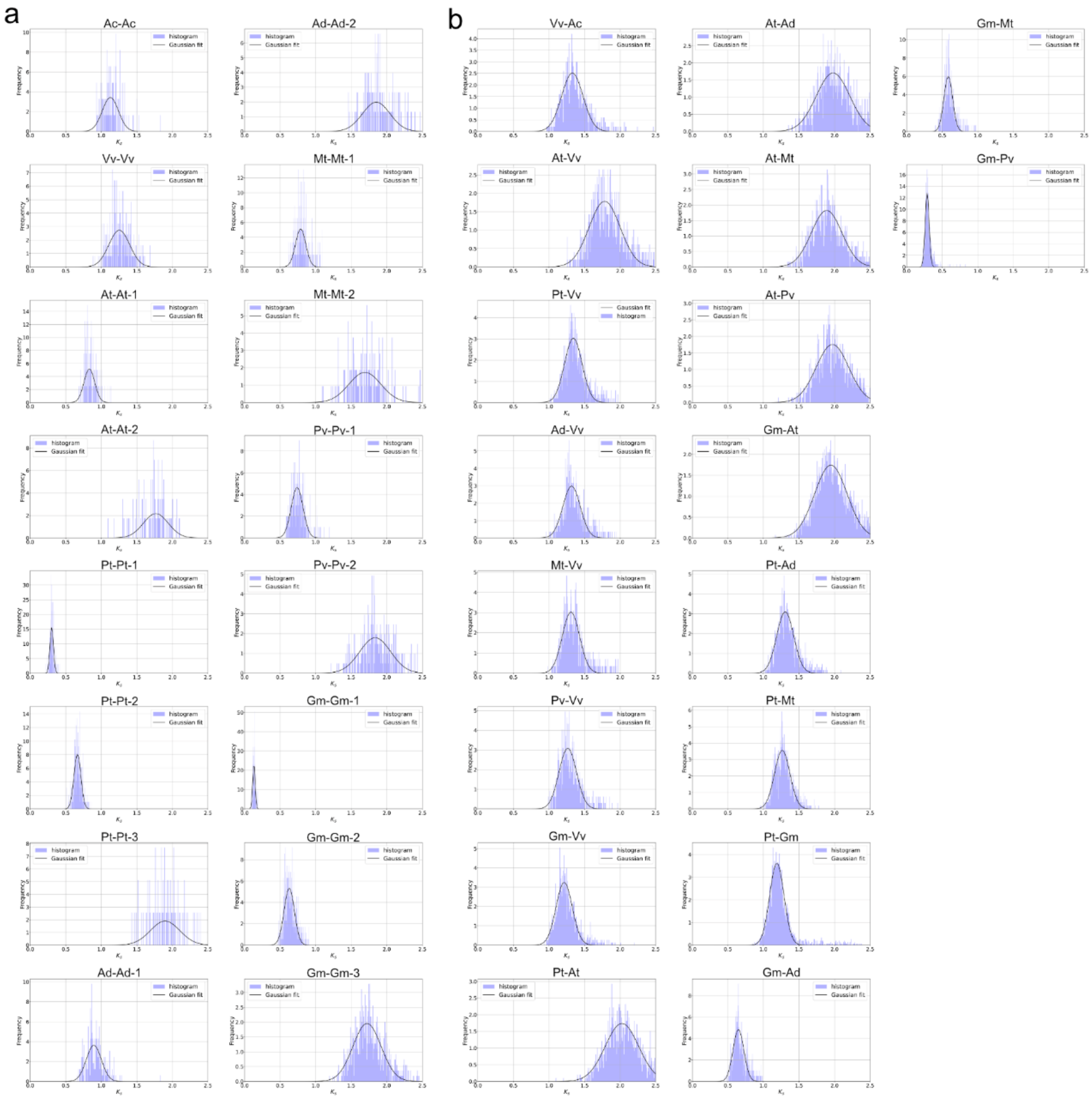

Supplementary Fig. 11 Histograms and fitted curves of synonymous nucleotide substitutions (*Ks*) of (a) intragenomic colinear homologous genes and (b) intergenomic colinear homologous genes. The median *Ks* value of each homologous block is used.

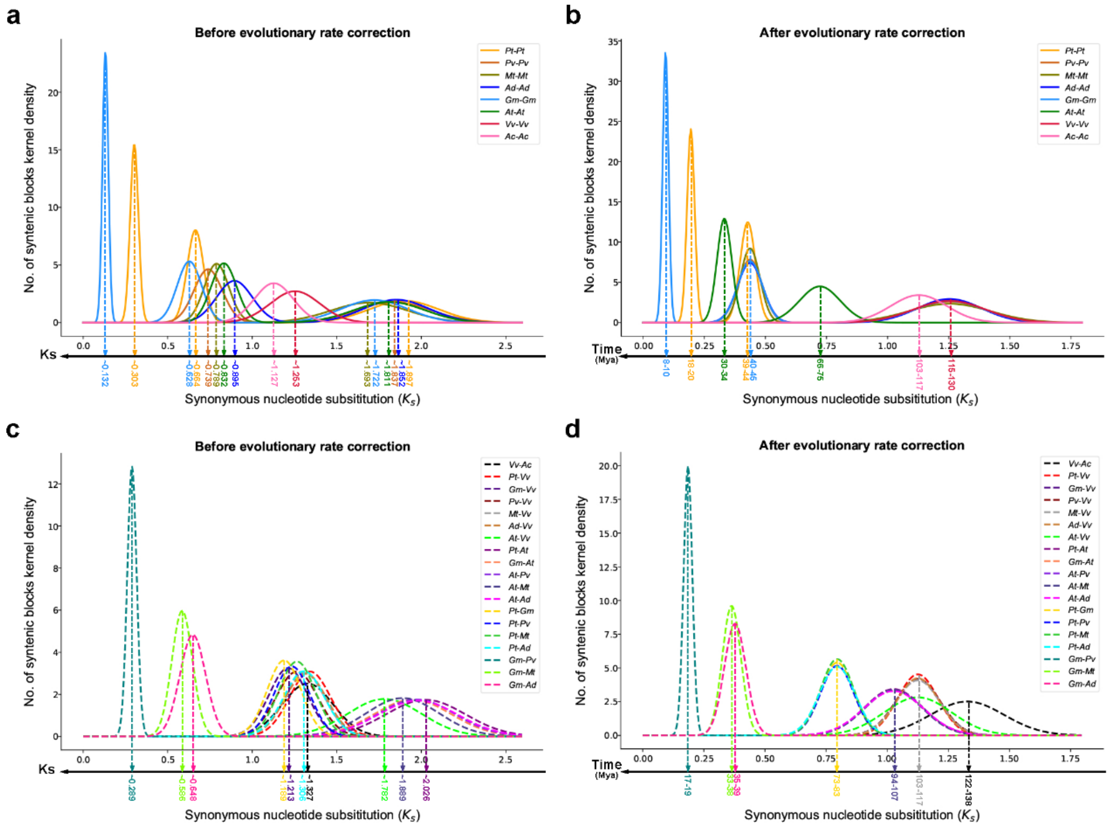

Supplementary Fig. 12 The original and corrected *Ks*, and inferred evolutionary dates among colinear genes. (a) *Ks* distribution and inferred evolutionary dates within a species before evolutionary rate correction. (b) *Ks* distribution and inferred evolutionary dates within a species after evolutionary rate correction (representing the polyploidization events). (c) *Ks* distribution and inferred evolutionary dates between any two species before evolutionary rate correction. (d) Ks distribution and inferred evolutionary dates between any two species after evolutionary rate correction (representing the speciation events).

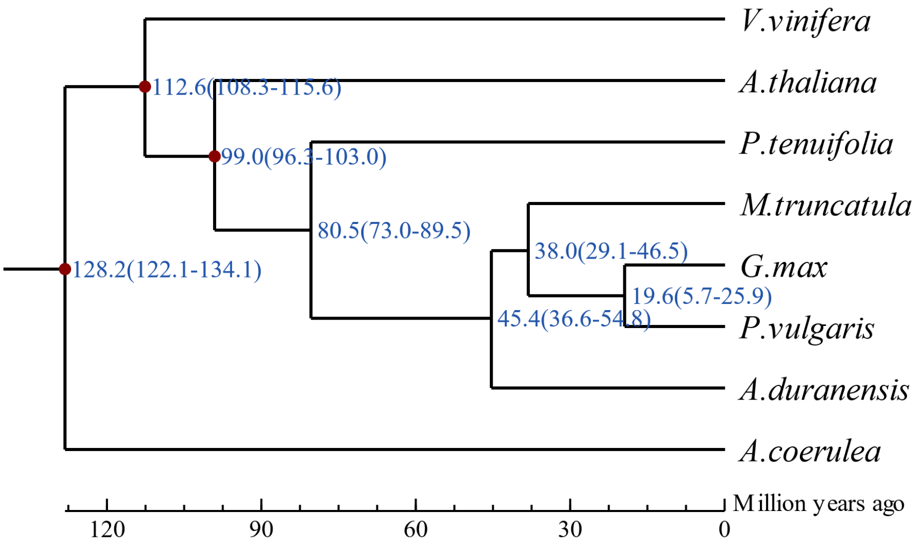

Supplementary Fig. 13 Phylogenetic tree and divergence time estimation of *P. tenuifolia* and the other 7 plant species. Divergence time was estimated using the mcmctree (http://abacus.gene.ucl.ac.uk/software/paml.html) embedded in the PAML package. The most likely divergence time from the most recent comment ancestor is given at each node, and the estimated ranges of divergence time are shown in the parentheses. Numbers in the parentheses are the predicted divergence time (95% confident intervals). The node dots indicate the calibration times of the divergence between *A. coerulea* and *A. thaliana* (122-134 Mya), *A. thaliana* and *V. vinifera* (105-115 Mya), *A. thaliana* and *P. vulgaris* (97-109 Mya). The calibration times were retrieved from the TimeTree database (http://timetree.org).

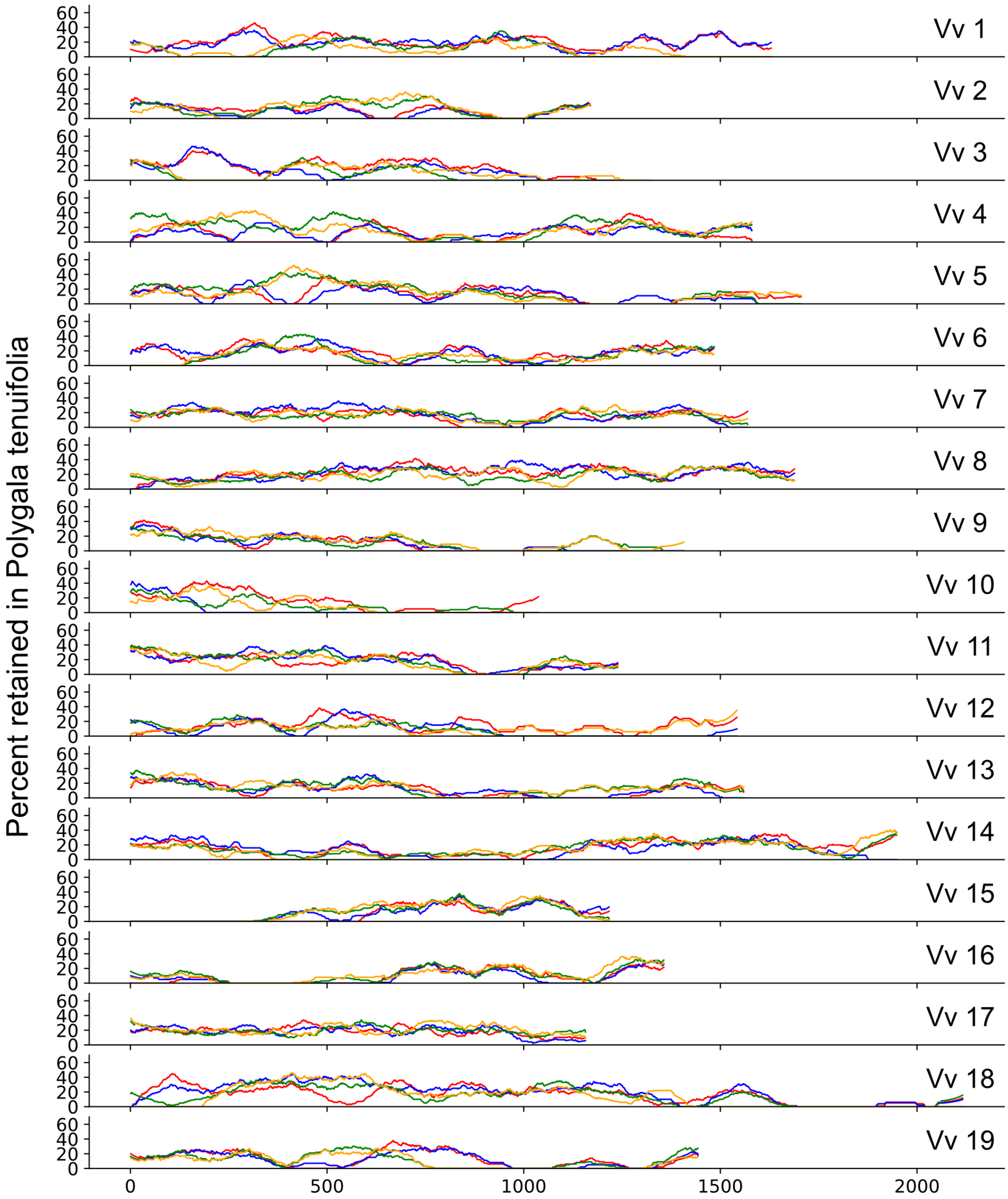

Supplementary Fig. 14 The retention of duplicated genes residing in four sub-genomes of *P. tenuifolia* using the grape as reference. The red, blue, green, and orange lines represent the retention of genes in the four sub-genomes (P11, P12, P21, and P22) of *P. tenuifolia*, respectively.

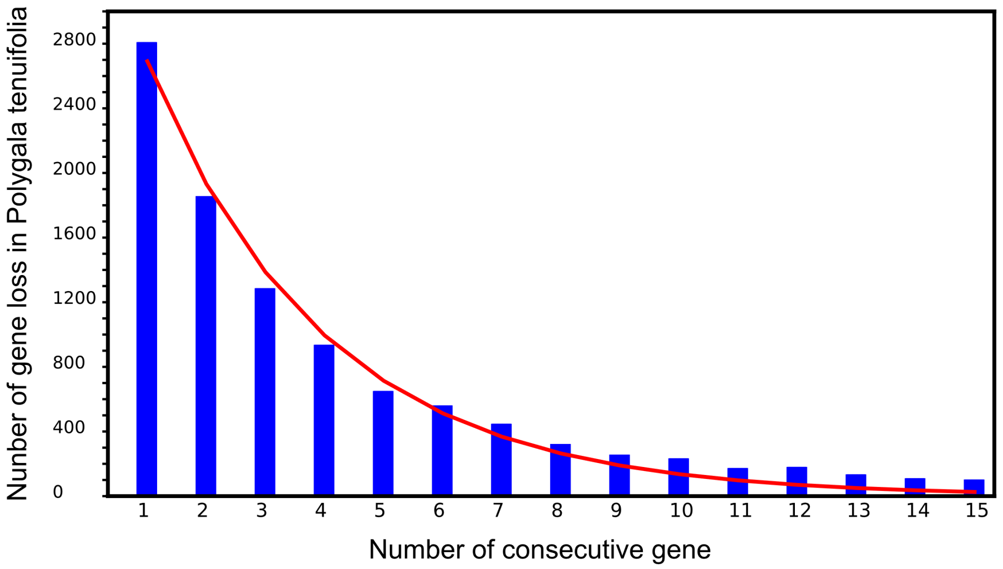

Supplementary Fig. 15 The geometric distribution of gene loss frequencies was fitted to *P. tenuifolia* using grape as reference.

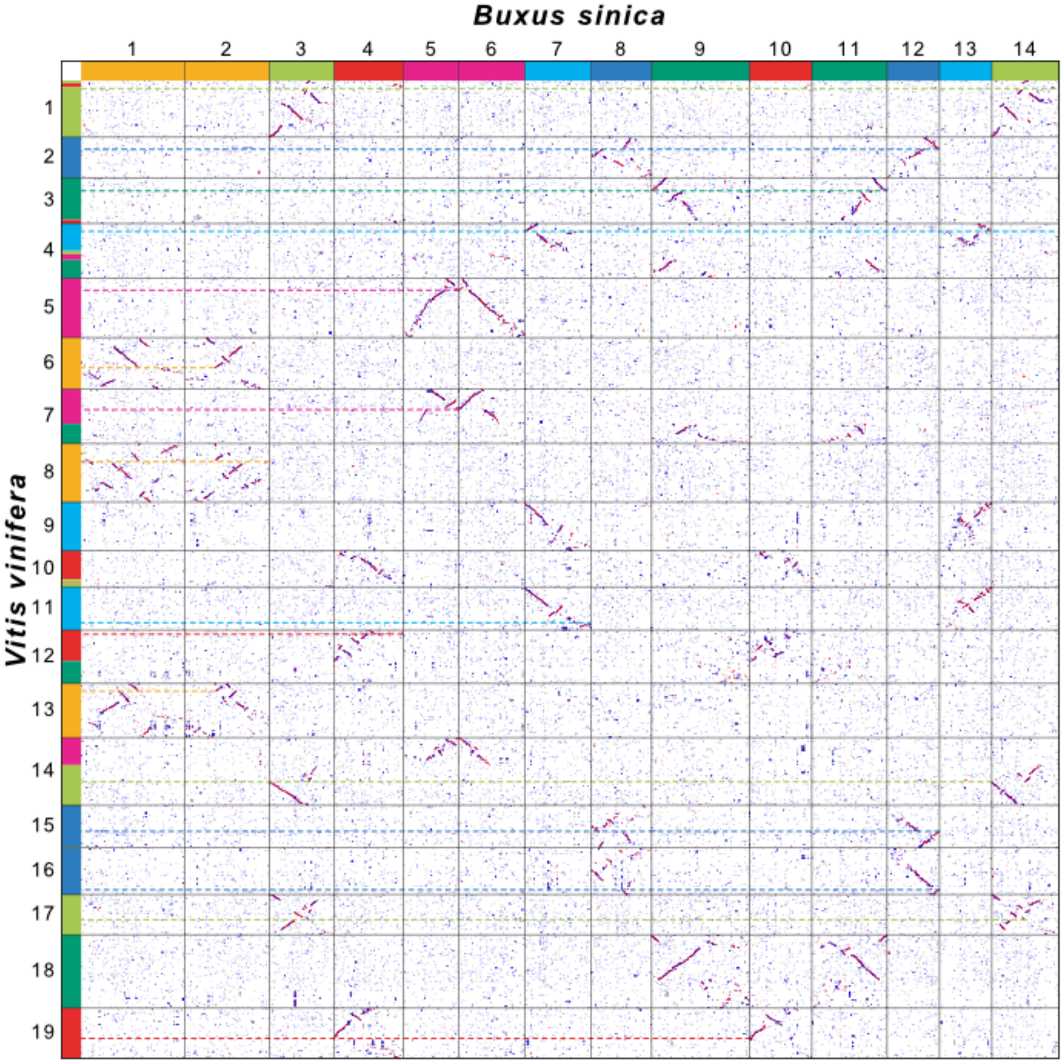

Supplementary Fig. 16 Homologous gene dot-plots and karyotype projections between grape and *Buxus sinica*. The dashed line indicates the position of the telomeres of the ancestral chromosome in the extant grape chromosome.

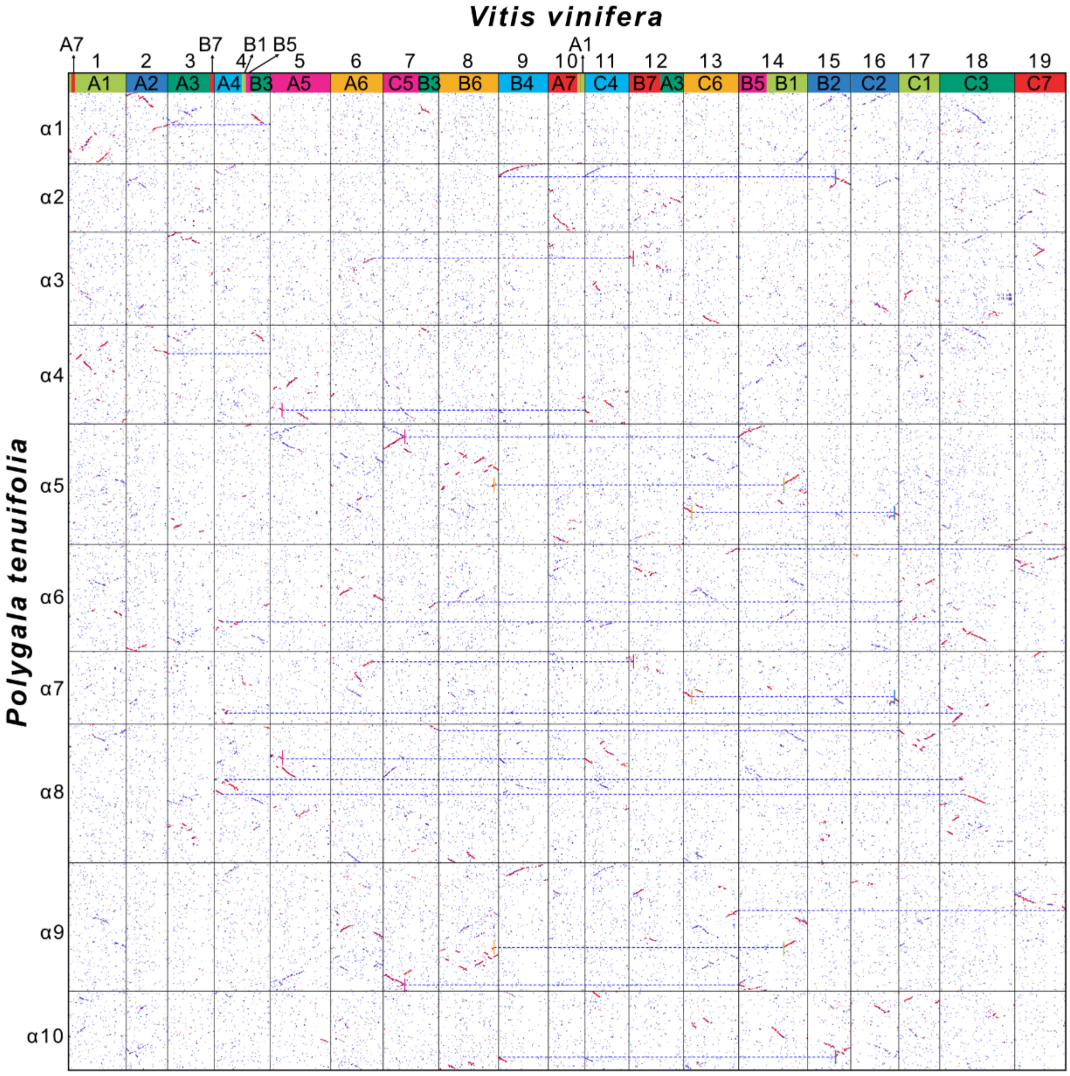

Supplementary Fig. 17 Homologous gene dot-plots and karyotype projections between pre-Pt-α ancestral genome and grape. The short vertical line indicates the position of the telomeres of the ancestral chromosome in the extant grape chromosome. The horizontal dashed line indicates that the telomeres of the ancestral chromosome fused in the pre-Pt-α ancestral chromosome.

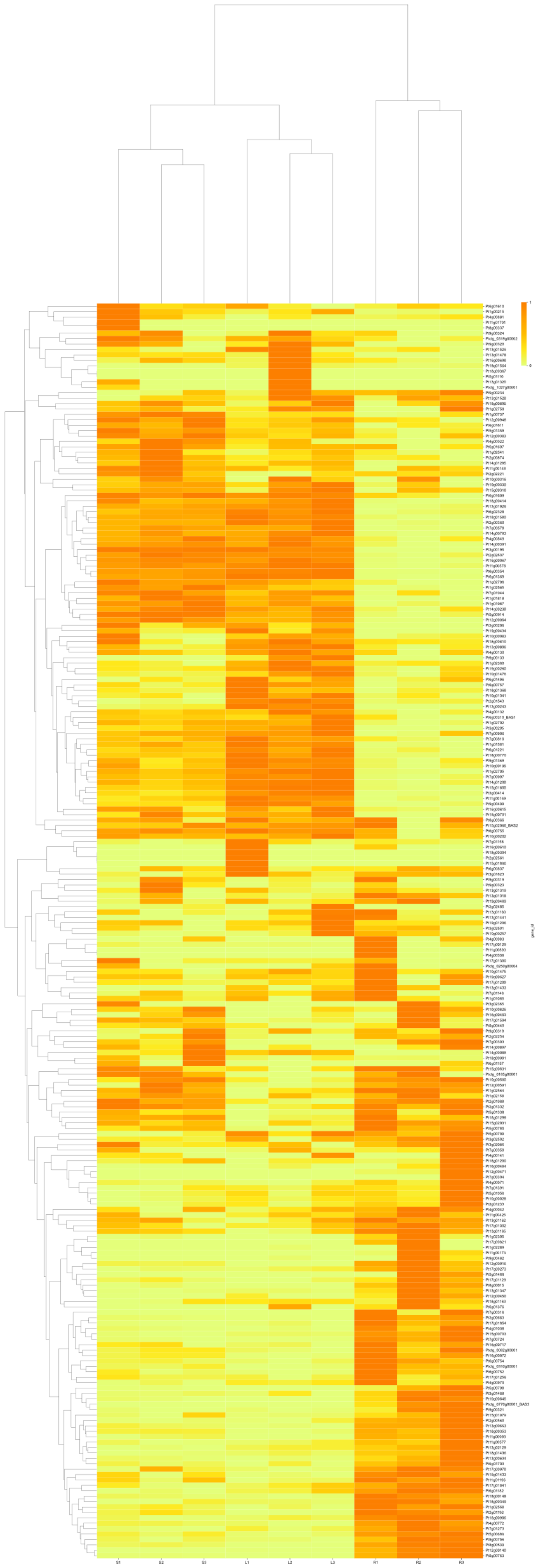

Supplementary Fig. 18 Expression pattern (TPM) of 248 P450 genes in roots (R1-R3), stems (S1-S3), and leaves (L1-L3) of *P. tenuifolia*. The heatmaps show hierarchical clustering of expression profiles with normalized expression levels.

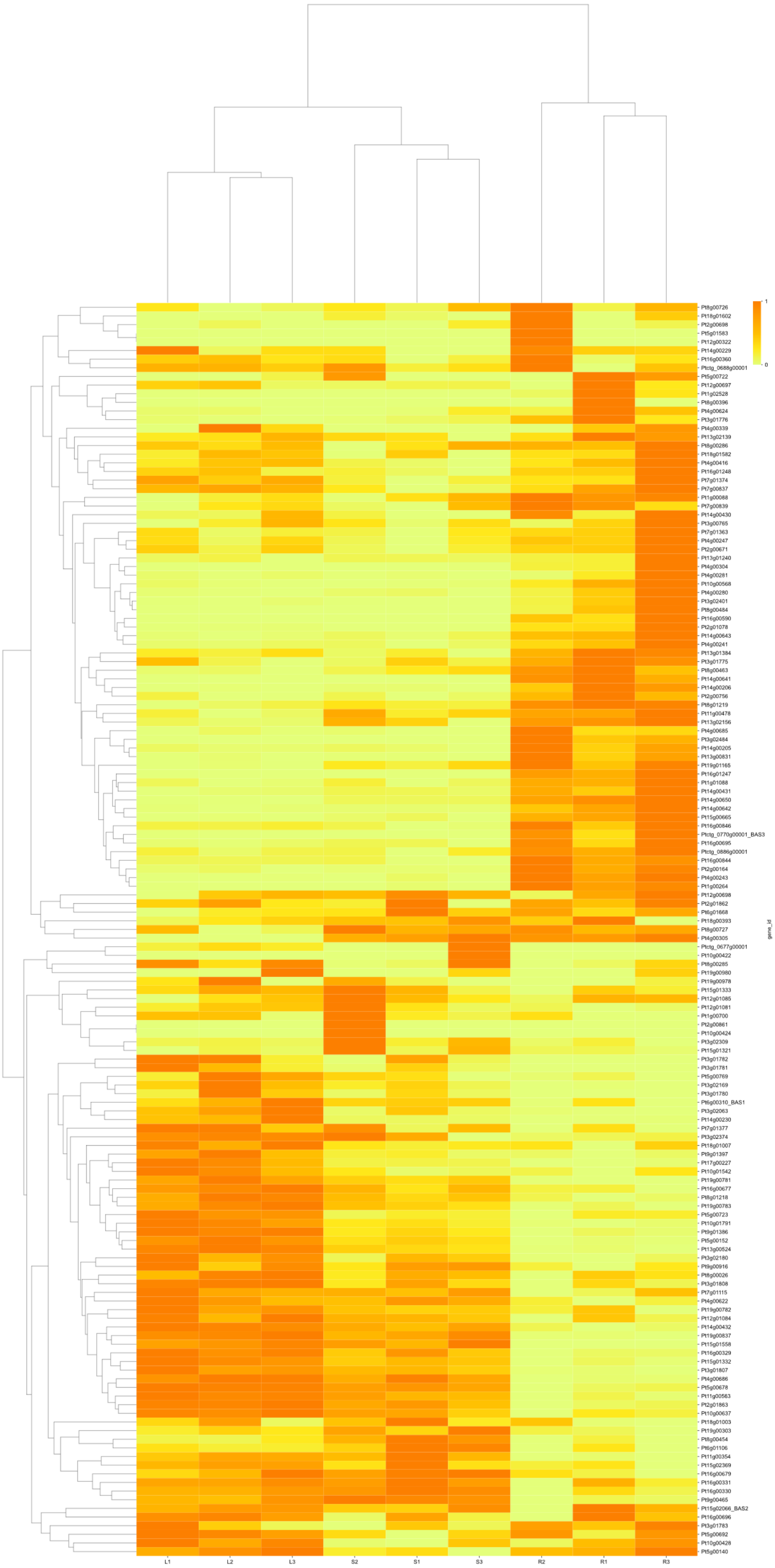

Supplementary Fig. 19 Expression pattern (TPM) of 152 UGT genes in roots (R1-R3), stems (S1-S3), and leaves (L1-L3) of *P. tenuifolia*. The heatmaps show hierarchical clustering of expression profiles with normalized expression levels.

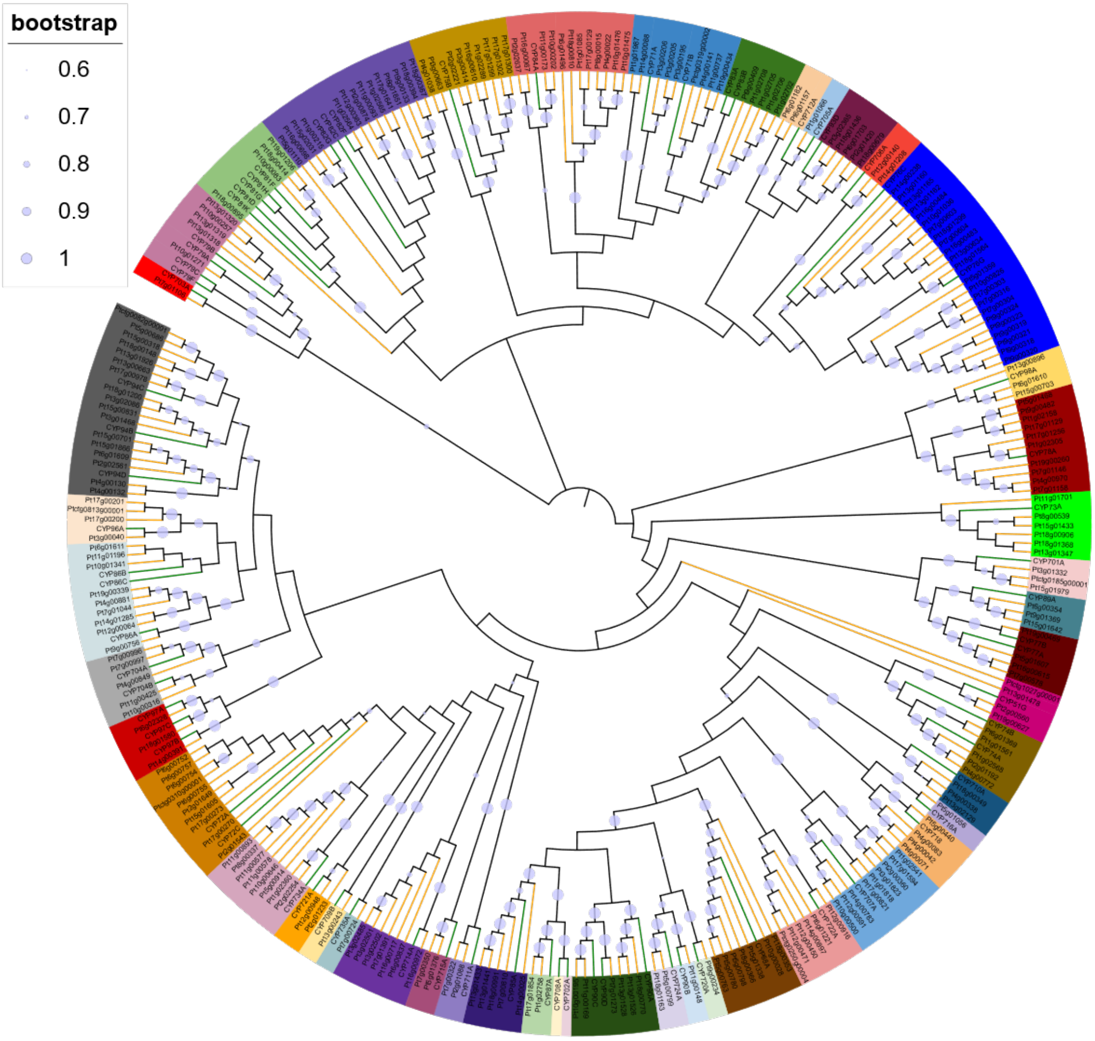

Supplementary Fig. 20 Phylogenetic analysis of P450 genes. The P450 gene families clustered into one clade are represented by different colors. The bootstrap value associated with each branch is represented by a light-purple circle: the larger the radius, the greater the bootstrap value.

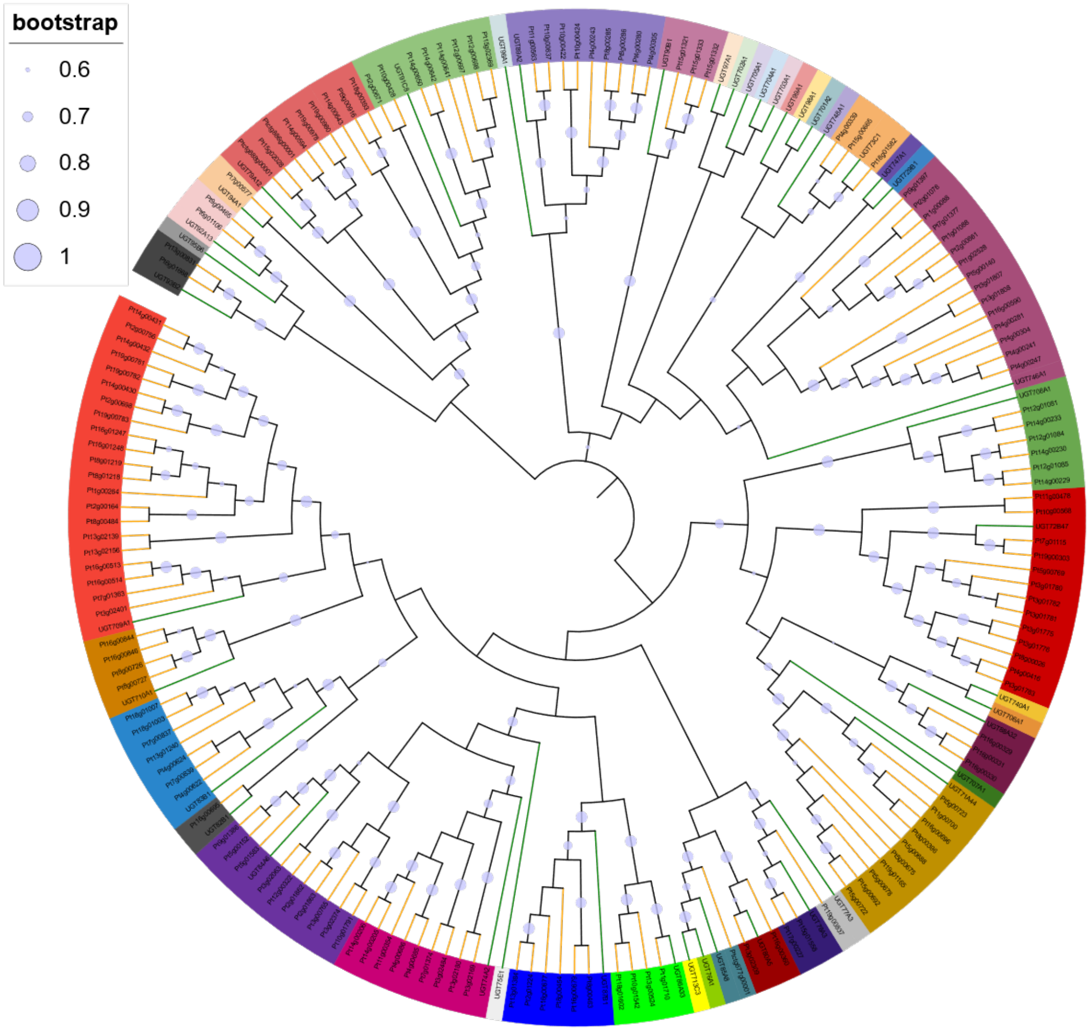

Supplementary Fig. 21 Phylogenetic analysis of UGT genes. The UGT gene families clustered into one clade are represented by different colors. The bootstrap value associated with each branch is represented by a light-purple circle: the larger the radius, the greater the bootstrap value.

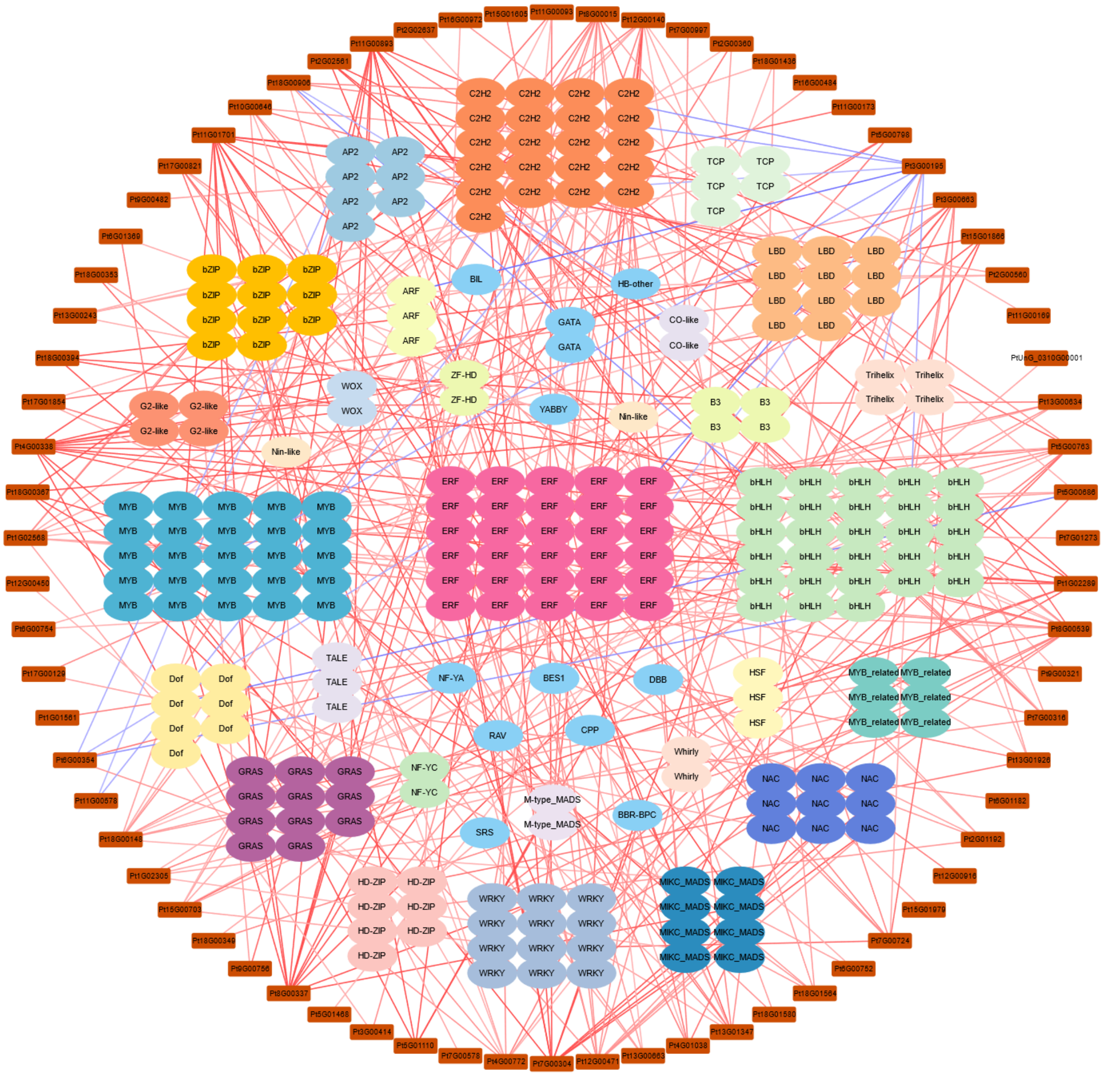

Supplementary Fig. 22 Correlation analysis between transcription factors and P450 genes. The outermost rectangle is the P450 genes. The oval in the circle is the transcription factors. Only the transcription factors and genes with the absolute value of correlation coefficients greater than 0.99 were shown and indicated by lines with different colors. The darker the color, the higher the correlation, red and blue represents positive and negative correlation, respectively.

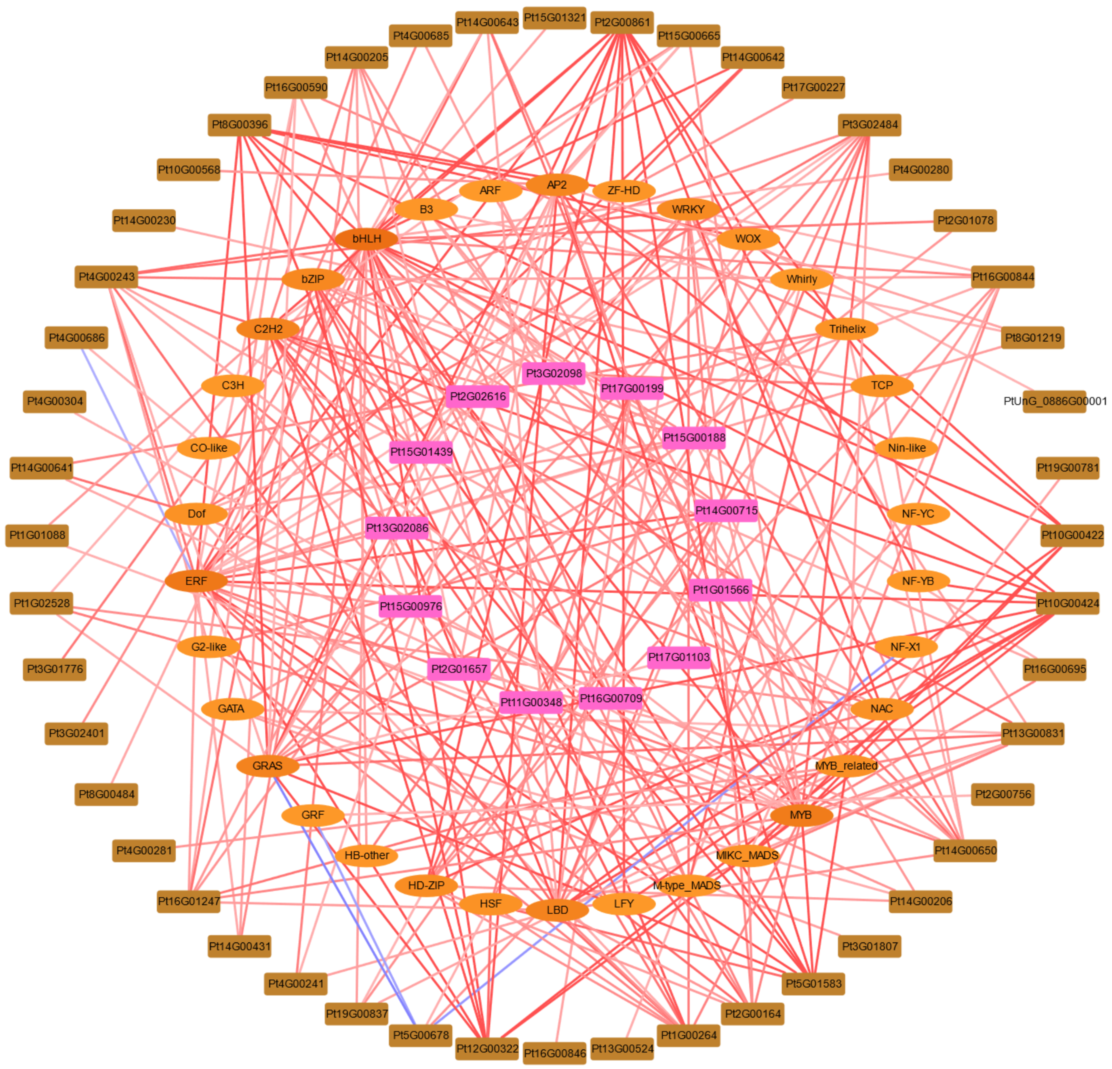

Supplementary Fig. 23 Correlation analysis of transcription factors with UGT and CSL genes. From the outer circle to the inner circle are UGT, transcription factors, and CSL, respectively. Only the transcription factors and genes with the absolute value of correlation coefficients greater than 0.99 were shown and indicated by lines with different colors. The darker the color, the higher the correlation, red and blue represents positive and negative correlation.
